# The Etiology of Advanced Chronic Kidney Disease in Southeast Asia: A Meta-analysis

**DOI:** 10.1007/s44197-024-00209-5

**Published:** 2024-04-08

**Authors:** Ni Made Hustrini, Endang Susalit, Felix Firyanto Widjaja, Anandhara Indriani Khumaedi, Olaf M. Dekkers, Merel van Diepen, Joris I. Rotmans

**Affiliations:** 1 Division of Nephrology and Hypertension, Department of Internal Medicine, Faculty of Medicine, Universitas Indonesia, Dr. Cipto Mangunkusumo National General Hospital, Jakarta, Indonesia; 2 Department of Internal Medicine, Leiden University Medical Center, Albinusdreef 2, Leiden, The Netherlands; 3 Diabetes Connection & Care, Eka Hospital Cibubur, Bogor, Indonesia; 4 Department of Clinical Epidemiology, Leiden University Medical Center, Leiden, The Netherlands

**Keywords:** Chronic kidney disease, Advanced-CKD, Primary kidney disease, Southeast Asia, Dialysis, Kidney transplantation

## Abstract

**Introduction:**

Chronic kidney disease (CKD) etiology varies greatly between developed and developing countries. In addition, differences in underlying pathogenesis and therapeutic options affect the progression towards advanced-CKD. This meta-analysis aims to identify the etiology of advanced-CKD in Southeast Asia.

**Methods:**

A systematic search in four electronic-databases and complementary search on national kidney registries and repository libraries was conducted until July 20, 2023. The risk of bias was assessed using Newcastle–Ottawa Scale for observational studies and Version-2 of Cochrane for intervention studies. A random-effects model was used to estimate pooled prevalence. The protocol is registered in the International Prospective Register of Systematic Reviews PROSPERO; Registration ID:CRD42022300786.

**Results:**

We analyzed 81 studies involving 32,834 subjects. The pooled prevalence of advanced-CKD etiologies are diabetic kidney disease (DKD) 29.2% (95%CI 23.88–34.78), glomerulonephritis 20.0% (95%CI 16.84–23.38), hypertension 16.8% (95%CI 14.05–19.70), other 8.6% (95%CI 6.97–10.47), unknown 7.5% (95%CI 4.32–11.50), and polycystic kidney disease 0.7% (95%CI 0.40–1.16). We found a significant increase in DKD prevalence from 21% (9.2%, 95%CI 0.00–33.01) to 30% (95%CI 24.59–35.97) before and after the year 2000. Among upper-middle-income and high-income countries, DKD is the most prevalent (26.8%, 95%CI 21.42–32.60 and 38.9%, 95%CI 29.33–48.79, respectively), while glomerulonephritis is common in lower-middle-income countries (33.8%, 95%CI 15.62–54.81).

**Conclusion:**

The leading cause of advanced-CKD in Southeast Asia is DKD, with a substantial proportion of glomerulonephritis. An efficient screening program targeting high-risk populations (diabetes mellitus and glomerulonephritis) is needed, with the aim to delay CKD progression.

**Supplementary Information:**

The online version contains supplementary material available at 10.1007/s44197-024-00209-5.

## Introduction

Chronic kidney disease (CKD) is a significant global health issue, leading to enormous morbidity and mortality. According to the recent Global Burden of Disease Study, there has been a significant increase in the rate of disability-adjusted life-years (DALYs) for CKD between 1990 and 2019, particularly in the age groups 50–74 years (from 1.6 to 2.3%; rank #14 to #8) and 75 years and older (from 1.6 to 2.5%; rank #14 to #9) [1]. In countries with lower and middle levels of socio-demographic index (SDI), the impact of age-standardised CKD is more pronounced, highlighting a significant disparity between the burden of CKD and the availability of adequate healthcare services [2]. A systematic review and meta-analysis conducted in 2016 investigated the worldwide prevalence of CKD based on stage, geographical location, gender and age group. The findings demonstrated a consistently high global CKD prevalence ranging from 11 to 13%, with the majority of cases being stage 3 (10.6%) [3]. Additionally, the study revealed that the CKD prevalence tend to increase with age, and is more common among females [3]. Despite developed regions displaying higher rates of CKD prevalence, the overall prevalence in developing areas was also notably high, ranging from 8.66 to 17.96% [3]. In 2017, approximately 844 million people worldwide were affected by CKD, with a higher burden observed among socially disadvantaged and vulnerable populations [4, 5]. While the impact of kidney disease is well-documented in developed countries, the magnitude of CKD in developing countries remains unclear [3–6]. A recent study revealed a wide variation in advanced-CKD (estimated glomerular filtration rate [eGFR] < 30 mL/min/1.73 m^2^) prevalence across Asian countries, with China and India having the highest proportion of adults with CKD [7].

In addition to the variation in the etiology of CKD among developed and developing countries,, the underlying pathogenesis of CKD differs as well [8]. Traditional risk factors such as diabetes mellitus and hypertension share an equal proportion as the leading causes of CKD, both in developed and many developing countries. However, glomerulonephritis is observed more frequent as cause of CKD in Asia and sub-Saharan Africa countries [8]. There are numerous factors considered to play a role in these differences in developing countries, particularly the constant predominant of infectious diseases in low-income countries, as a consequence of poor sanitation, inadequate access to safe water, and high prevalence of disease-transmitting vectors [8, 9]. Furthermore, the influence of environmental pollution, pesticides, analgesic abuse, herbal medications, and use of unregulated food additives also plays a role in the development of CKD in developing countries [8–11]. However, changes in lifestyles, rapid urbanization and globalization in developing countries have augmented the demographic transition, which has directed to an intersection of disease burdens, where prevalence of infectious diseases remains high, yet prevalence and severity of metabolic disorders, such as diabetes and hypertension, increases [8]. Geographically, Southeast Asia is situated in a region that is highly susceptible to natural disasters such as earthquakes, floods, and environmental pollutions which could potentially impact the population’s health, including kidney health through the development of acute kidney injury (AKI)-associated CKD [12]. Moreover, Southeast Asia exhibits not only geographical diversity but also significant variation in socio-cultural and economic status. The included countries vary greatly in terms of wealth, ranging from very rich to extremely poor [13]. These factors have resulted in distinct health statuses and healthcare systems both within and between countries.

These settings emphasize the urgent need for early detection to target better prevention and treatment of CKD in Asia. However, the underlying etiologies of CKD have not been clearly identified in most regions in Asia, particularly in Southeast Asia. Therefore, this meta-analysis aims at providing summarized evidence on the underlying kidney disease in advanced CKD including those receiving kidney replacement therapy (KRT) in Southeast Asia.

## Methods

### Eligibility Criteria and Search Strategy

We conducted this meta-analysis following the Meta-analysis of Observational Studies in Epidemiology (MOOSE) reporting guidelines [14] (Supplementary Form 1), and the review protocol was registered on the International Prospective Register of Systematic Reviews (PROSPERO; Registration ID: CRD42022300786).

The search strategy was systematically conducted across several electronic databases: PubMed, Embase, Cochrane Library, and Scopus, from September 1 to November 10, 2022. In addition, we complemented the initial search strategy by adding potentially relevant studies from the national kidney registries and repository libraries until July 20, 2023. The search conducted on the Indonesia’s kidney registries and repository libraries included the use of Bahasa Indonesia language.

We included observational and interventional studies from the 11 countries in Southeast Asia, that is Brunei Darussalam, Cambodia, East Timor, Indonesia, Laos, Malaysia, Myanmar, the Philippines, Singapore, Thailand, and Vietnam. We included published studies that describe the underlying kidney disease in patients with advanced CKD, articles published in English or Bahasa Indonesia, as well as studies involving dialysis (hemodialysis/HD or peritoneal dialysis/PD) and kidney transplantation patients. We excluded articles from the meta-analysis if they were not original investigations, such as reviews, reports from conference abstracts, case reports/series, and study protocols. Additionally, any studies involving patients under 15 years old, individuals without chronic kidney disease, or those that included subjects that could potentially influence the distribution of the underlying kidney disease (e.g., studies that only recruited selected CKD population such as limited to lupus nephritis or diabetes population) were also excluded. In cases where multiple studies used the same cohort dataset, we selected the one that provided the most information to use in our analysis.

The search was not restricted to a specific publication year. NH independently performed the database search, following the guidance of expert librarians, using search terms related to the patient population of interest, specifically chronic kidney disease and its stages, countries in the Southeast Asia region, and kidney replacement therapies such as dialysis (hemodialysis or peritoneal dialysis) and kidney transplantation. The complete search strategy can be found in Supplementary Table S1.

### Definition of Variables and Outcomes

Chronic kidney disease was defined as any abnormalities in kidney structure or function (eGFR < 60 ml/min/1.73 m^2^) lasting for at least three months, with implications for health [15]. Advanced CKD was defined as eGFR < 30 ml/min/1.73 m^2^ (KDIGO stage 4 and 5). The primary kidney disease was categorized into six different etiologies based on KDIGO criteria, that is diabetic kidney disease (DKD), hypertensive nephrosclerosis, glomerulonephritis, polycystic kidney disease (PKD), other, and unknown.

### Data Extraction and Risk of Bias Assessment

Two researchers (NH, AI) independently reviewed titles and abstracts according to our in- and exclusion criteria. Any disagreements between the two researchers were resolved through consensus or by involving a third researcher (FW). The interrater reliability (IRR) analyses between the three researchers was conducted in two stages: initially and midway through the screening process (Supplementary Fig. 1). The intraclass correlation (ICC) statistics were used to determine the degree of correlation and agreement between the measurements of each researcher. We observed excellent reliability in the IRR assessment during both stages, with confidence intervals of 0.989–0.991 and 0.979–0.985 for the ICC (Supplementary Table S2).

Two authors (NH and FW) independently gathered data from each included study. We collected the following information: the first author and year of publication, country where the study was conducted, study design, primary outcome, the stage of chronic kidney disease (CKD), form of kidney replacement therapy (hemodialysis, peritoneal dialysis, kidney transplantation), the study population, sample size, study duration, demographic information (mean age, percentage of male/female sex), and the distribution of the primary cause of kidney disease.

Risk of bias assessment was conducted by two authors (NH and FW) independently. We used the Newcastle–Ottawa Quality Assessment Scale for observational studies (cross-sectional, cohort, and case–control) to evaluate the studies. A combination of seven and eight multiple-choice questions were used to score the studies in three areas: (1) selection of study participants (maximum of 5 stars in cross-sectional studies and 4 stars in cohort and case–control studies); (2) comparability between groups (maximum of 2 stars); (3) outcomes (maximum of 3 stars in cross-sectional studies and cohort) and exposure in case–control studies (maximum 3 stars). A total score of less than 3 indicates a high risk of bias, 4–6 indicates a moderate risk, and 7–9 indicates a low risk. For randomized trials, the Version-2 of the Cochrane risk of bias tool is used to assess potential bias. For non-randomized intervention studies, the Risk of Bias in Non-Randomized studies tool is used to evaluate bias.

### Synthesis Methods and Data Analysis

The primary outcome was the overall distribution of primary renal disease in Southeast Asian countries. Our secondary outcomes include stratifications by country, time period (i.e., before and after the year 2000) and economic status according to the World Bank (i.e., high-income, upper-middle-income, and lower-middle-income country) [16]. Studies that report more than six different underlying kidney diseases will be categorized according to the mentioned etiology classification. The proportions were transformed using the Freeman-Tukey double arcsine transformation to calculate weighted pooled estimates. This provides confidence intervals for the pooled estimates and tests for significance based on normal approximation and variance stabilization. The heterogeneity of the studies was measured using the *I*^*2*^ statistics, where an *I*^*2*^ value greater than 50% indicates significant heterogeneity.

A sensitivity analysis, we repeated our main meta-analysis including only low risk of bias studies to assess robustness of the pooled distribution of CKD etiology.

We conducted our meta-analysis using *metaprop* in STATA software version 14 (StataCorp, Texas, USA).

## Results

### Characteristics of Study Population

**Fig. 1:**
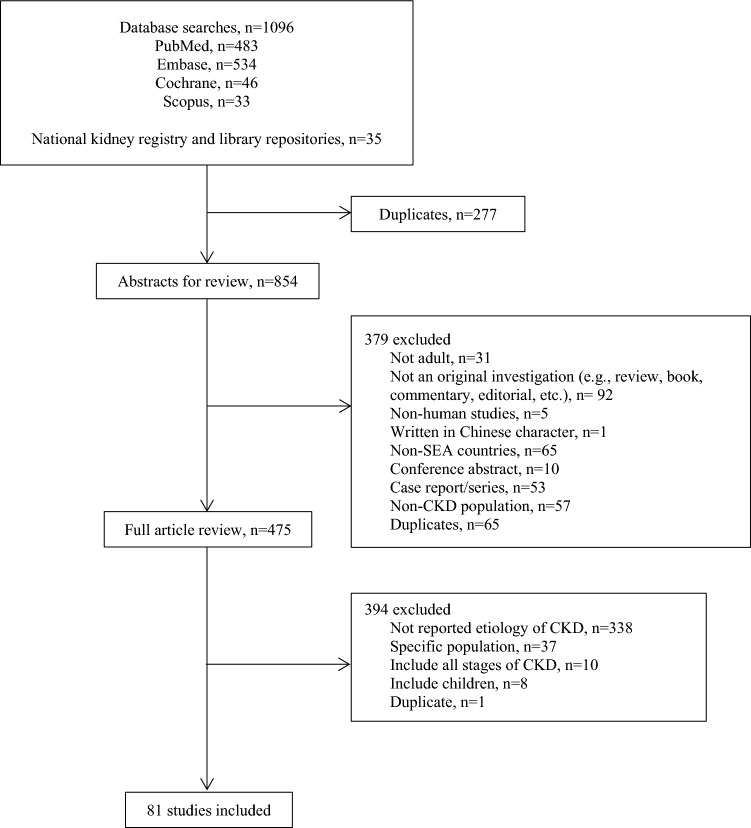
Selection process for the included studies

We identified 1131 studies, of which 1096 were retrieved from database searches and 35 from national kidney registry and library repositories. After the initial screening process, 475 studies were eligible for full-text review. Of these, 81 studies met the inclusion criteria, involving 32,834 participants (Fig. 1. Selection of the included studies). Two countries, East Timor and Laos, did not have any available articles among the eleven countries in the region. Our analysis included 75 observational studies (38 cohort, 35 cross-sectional, 1 cross-sectional & case–control, and 1 cross-sectional & cohort study), 3 randomized controlled trials, and 3 registry data. The majority of the reports came from five countries: Thailand (21 studies, 25.9%; n = 12,215), Malaysia (19 studies, 23.5%; n = 3378), Singapore (18 studies, 22.2%; n = 13,013), Indonesia (11 studies, 13.6%; n = 1805), and Brunei Darussalam (5 studies, 6.2%; n = 1105). These studies were published between 1986 and 2022 in high-income to lower-middle-income countries (Table 1). The study population mainly consisted of dialysis patients (n = 17,533, 53%) and kidney transplant recipient (n = 9722, 30%), while the smallest proportion originated from the non-dialysis population (n = 1379, 4.2%) (Supplementary Table S3). Detailed characteristics of all included studies are presented in Table 2. The assessment of bias in the included studies (excluding three registry studies) showed that less than half of the studies (n = 34) had a low risk of bias.

**Table 1:** Summary of included studies

Country	Economic status	Number (%) of studies	Publication period	Number of participants
Brunei Darussalam	High-income	5 (6.17%)	2008–2017	1105
Cambodia	Lower-middle-income	1 (1.23%)	2022	407
Indonesia	Upper-middle-income	11 (13.58%)	2017–2022	1805
Malaysia	Upper-middle-income	19 (23.46%)	1986–2022	3378
Myanmar	Lower-middle-income	1 (1.23%)	2020	54
Philippine	Lower-middle-income	3 (3.70%)	2010–2022	548
Singapore	High-income	18 (22.22%)	1991–2020	13,013
Thailand	Upper-middle-income	21 (25.93%)	1999–2022	12,215
Vietnam	Lower-middle-income	2 (2.47%)	2015–2019	309
Total		81 (100%)		32 834

**Table 2:** Characteristics of the included studies

No	Author	Country	Type of study	Primary outcome	Study size	Study population	Age (year)	Male (%)	Risk of bias
1	Bavanandan, et al. (2015)	Malaysia	Cross-sectional	Costs and utility of transplantation, derived from the results of survival and quality of life analysis	109	Kidney transplant	Mean (SD): LKT: 33.4 (10.3) DKT: 41.8 (8.9)	60.55%	Low
2	Bawazier, et al. (2018)	Indonesia	Cohort	The each groups’ mean score of quality of life dimensions	39	Hemodialysis	n (%): < 35 years: 3 (7.7) 35–60 years: 29 (74.4) > 60 years: 7 (17.9)	46.15%	High
3	Bawazier, et al. (2019)	Indonesia	Cross-sectional	The mean QoL of each group (dialyzer reuse group and single-use dialyzer group)	389	Hemodialysis	Mean (SD): 49.3 (11.5)	57.58%	Low
4	Boongird, et al. (2022)	Thailand	Cohort	To evaluate SARS-CoV-2-specific humoral and cell-mediated immune responses following two doses of the inactivated whole-virus SARS-CoV-2 vaccine, with a 4-week interval between doses, in patients with ESKD receiving dialysis and compared to healthy individuals	60	Hemodialysis and peritoneal dialysis	Mean (SD): HD: 45 (10) PD: 41 (11)	66.67%	Low
5	Bunchorntavakul, et al. (2014)	Thailand	Cross-sectional	To evaluate the prevalence of UGI lesions and H. pylori in non-dyspeptic KT candidates, as well as to determine the factors significantly associated with significant EGD findings and H. pylori infection	107	Kidney transplant	Mean (SD): 39.4 (10.3)	53.27%	Low
6	Bunnag, et al. (2011)	Thailand	Cross-sectional	To evaluate the risk factors of intraperitoneal infection in the CAPD patients in Rajavithi Hospital	27	Peritoneal dialysis	Mean (SD): 59.7 (14.5)	59.26%	Low
7	Chan, et al. (2019)	Malaysia	Non-randomized, single arm community trial	To investigate the efficacy of the patient educational program (PEP) in optimal phosphate control among hemodialysis patients	57	Hemodialysis	Mean (SD): 54 (13)	42.11%	Moderate
8	Chan, et al. (2012)	Malaysia	Cross-sectional	To determine the overall compliance behaviour to therapeutic regimens among patients undergoing hemodialysis and to determine the factors contributing to compliance among these subjects	188	Hemodialysis	Mean (SD): 58.2 (10.5)	48.94%	Low
9	Cheung, et al. (2012)	Singapore	Cohort	To assess the measurement properties of the Chinese version (simplified characters) of the KDQOL-SF	78	CKD5	Median (range): 76 (52–98)	55.13%	Moderate
10	Chiasakul, et al. (2015)	Thailand	Cohort	CMV infection	121	Kidney transplant	Mean (SD): 43 (12)	47.93%	Low
11	Chitasombat, et al. (2018)	Thailand	Cohort	The burden of symptomatic CMV reactivation/disease following the use of ATG in situations where CMV prophylaxis was not widely available and affordable	30	Kidney transplant	Median (range): 51 (25–68)	43.33%	Low
12	Chittinandana. (1999)	Thailand	Registry	Registry report	1802	ESKD on RRT	Mean (SD): 53 (15)	54.16%	Not assessed
13	Chong, et al. (2008)	Brunei Darussalam	Cohort	The incidence of HCV infections and looked at the characteristics and outcomes among patients undergoing HD	71	Hemodialysis	Mean (SD), range: 48 (14.7), 17–72	52.11%	Moderate
14	Chong, et al. (2013)	Brunei Darussalam	Cross-sectional	To assess the prevalence of GI symptoms among patients with ESRD undergoing regular hemodialysis (HD) in an Asian population	123	Hemodialysis	Mean (SD): 51.8 (12.9)	47.15%	Moderate
15	Choo, et al. (2012)	Singapore	Cohort	To report clinical characteristics including the relationship between causes of ESRD namely DM and GN on survival outcomes of patients initiated on PD between 2000 and 2008 in the SGH	1,015	Peritoneal dialysis	Mean (SD): 58 (12.4)	52.71%	Moderate
16	Choong, et al. (1991)	Singapore	Cohort	The survival, causes of mortality and some complications occurring in these three groups of patients (Centre Dialysis, Home Dialysis and Self -Dependency Dialysis) while on maintenance haemodialysis from 1968 to 1989	425	Hemodialysis	Mean (SD): 36 (9)	52.47%	Moderate
17	Danguilan, et al. (2010)	Philippine	Cohort	To assess the safety and effectiveness of subcutaneous (SC) Epoetin alfa in the treatment of Filipino patients with anemia associated with chronic renal failure	458	CKD pre- and on-dialysis	Mean (SD): 52.8 (16.9)	50.22%	High
18	Disthabanchong, et al. (2018)	Thailand	Cohort	Examined the abilities of AAC and PAC on lateral abdominal and pelvic radiographs in predicting mortality in three groups of CKD population: non-dialysis CKD stages 2–5 (CKD 2–5), maintenance hemodialysis (HD) and long-term KT (KT) patients	259	Hemodialysis and kidney transplant (we exclude CKD2-5 group in analysis)	Mean (SD): HD: 49.5 (13.2) KT: 48.7 (11.6)	55.60%	Low
19	Duong, et al. (2015)	Vietnam	Cross-sectional	The point seroprevalence, risk factors and genotype of HBV and HCV in newly admitted ESRD patients	113	Hemodialysis	Mean (SD): 53 (16)	47.79%	Moderate
20	Goh, et al. (2002)	Singapore	Cross-sectional	The profile of admissions to the acute dialysis care facility	119	CKD5 (we exclude acute renal failure group in analysis)	None	None	Moderate
21	Griva, et al. (2017)	Singapore	RCT	Serum potassium and phosphate concentrations and interdialytic weight gains (IDWGs)	235	Hemodialysis	Mean (SD): 53.5 (10.4)	58.30%	Low
22	Griva, et al. (2018)	Singapore	Cross-sectional	Examined unintentional, intentional and overall self-reported non-adherence in renal transplant recipients, and the associations between these behaviours and socio-demographic and disease/treatment characteristics, and several psychosocial factors, including beliefs about medicines, social support, and emotional distress	152	Kidney transplant	Mean (SD): 49.45 (11.44)	50.66%	Moderate
23	Hieu, et al. (2019)	Vietnam	Cross-sectional and case-controlled	To investigate the frequencies of each allelic group, genotype, and haplotype in the genetic loci of HLA class I (HLA-A, -B) and class II (HLA-DRB1) in Vietnamese patients with ESRD and to determine which HLAs are associated with ESRD	196	Kidney transplant	Mean (SD): 37.81 (11.80)	75.00%	Moderate
24	Htun, et al. (2020)	Myanmar	Cross-sectional	To determine the frequency of the CYP3A5*3 genetic polymorphism and its effect on the pharmacokinetics of tacrolimus in renal transplant recipients	54	Kidney transplant	Mean (SD): CYP3A5 non-expressor: 39.9 (12.1) CYP3A5 expressor: 38.2 (10.9)	66.67%	Low
25	Hyodo T, et al. (2022)	Cambodia	Cross-sectional	To improve current knowledge of HD patients and thus improve their outcomes and develop better approaches for this population	407	Hemodialysis	Mean (SD): 52 (15)	57.25%	High
26	Ingsathit, et al. (2010)	Thailand	Cohort	To determine kidney allograft survival, and the causes of graft loss and death in Thai patients in the past decade	2298	Kidney transplant	Mean (SD): 41.7 (12.4)	59.75%	Low
27	Ingsathit, et al. (2013)	Thailand	Cohort	To evaluate the long-term outcomes of kidney re-transplantations compared with first kidney transplantations	116	Kidney transplant	Mean (SD): 46.2 (12.8)	68.97%	Moderate
28	Jalalonmuhali, et al. (2020)	Malaysia	Cross-sectional and cohort	Aims to determine the prevalence of AT1R-Ab among the Malaysian population	99	Kidney transplant	Mean (SD): AT1RAb(-): 43.35 (11.72) AT1RAb(at risk): 40.11 (10.64) AT1RAb( +): 34.73 (8.34)	55.56%	Moderate
29	Jonny, et al. (2020)	Indonesia	Cross-sectional	To describe our experience on a simple technique of Tenckhoff catheter insertion by a nephrologist called the Bandung method	230	Peritoneal dialysis	Mean (SD): 47.28 (14.94)	58.70%	Moderate
30	Koniman, et al. (2020)	Singapore	Cohort	Early technique failure, which was defined as transfer to HD for ≥ 30 days or death, within the first year of PD initiation	517	Peritoneal dialysis	Mean (SD): 64.5 (13.6)	45.07%	Low
31	Lee, et al. (2017)	Singapore	Cohort	To evaluate the outcomes of AVF creation, effect of preoperative vein mapping and predictors of fistula success in our incident haemodialysis patients	694	Hemodialysis	Median (IQR): 64 (55–72)	62.25%	Low
32	Lew, et al. (2017)	Singapore	Cohort	To build a population-based cohort of Chinese for long-term study of dietary, genetic and environmental determinants of cancer and other chronic diseases in Singapore	822	General population	None	None	Low
33	Liu, et al. (2007)	Malaysia	Cross-sectional	Analysis of all newly diagnosed ESRD patients in HSAJB prospectively registered from 1stJanuary 2003 to 31st December 2004	605	CKD5	Mean (SD): 2003: 50.8 (15.1) 2004: 50.8 (15.1)	50.58%	High
34	Loy, et al. (2013)	Singapore	Cohort	To investigate the risk of cancer in ESRD patients on dialysis	5505	CKD 5D	Mean (SD): 58.1 (13.0)	52.23%	Moderate
35	Lumlertgul, et al. (2017)	Thailand	Cross-sectional	To determine prevalence of abdominal aortic calcification (AAC) detected by plain lateral abdominal radiograph	686	CKD 5D	Median: 54–60 Median (range): CKD3-5ND: 60 (52–65) CKD5D: 54 (43–61)	53.50%	Low
36	Luvira, et al. (2011)	Thailand	Cohort	To identify the incidence and epidemiological data of CAPD-related infection	333	Peritoneal dialysis	Mean (SD): 57.2 (15.4)	60.96%	Low
37	Malaysian Dialysis and Transplant Registry. (2021)	Malaysia	Registry	Registry report	78	Kidney transplant	Mean (SD): 38 (12)	0.63%	Not assessed
38	Marbun, et al. (2017)	Indonesia	Cohort	To determine the survival of graft and patient in the first 3 years after transplantation and at the end of study	138	Kidney transplant	Mean (SD): 47,94 (14,06)	70.29%	Moderate
39	Maulidya, et al. (2022)	Indonesia	Cross-sectional	To describe the iron status of patients with chronic kidney disease stage 5 undergoing regular hemodialysis	80	Hemodialysis	Mean: 46.75 Median (range): 45 (18–71)	45.00%	High
40	Mok, et al. (2012)	Singapore	Cohort	Analyses the outcomes of kidney transplant recipients	450	Kidney transplant	Mean (SD): DD: 45.7 (8.2) LD: 37.1 (11.8)	50.89%	Low
41	Ng, et al. (2012)	Malaysia	Cohort	To describe the experience and the results of our IPD program as a viable interim option to an eventual definitive RRT	39	Peritoneal dialysis	Mean (SD): 54.6 (11.6)	46.15%	Moderate
42	Noppakun, et al. (2015)	Thailand	Cohort	Graft loss that was defined as return to dialysis, graft removal, retransplant, or patient death	3973	Kidney transplant	Mean (SD): 42.2 (13.0)	62.09%	Moderate
43	Ong, et al. (2017)	Philippine	Cross-sectional	To evaluate the compliance of HD patients’ to RRT in two dialysis centers in Iloilo City	61	Hemodialysis	Mean (SD): 47.57 (12.41)	42.62%	Moderate
44	Paloyo, et al. (2022)	Philippine	Cohort	Report of initial experience of the Z Benefit Package	29	Kidney transplant	Median (range): 34 (17–58)	55.17%	Moderate
45	Panaput, et al. (2022)	Thailand	Cohort	Disease-related death (included all mortalities excluding any death from accident) and the first hospitalization of any cause	407	Hemodialysis	Mean (SD): 54.8 (13.5)	56.76%	Low
46	Phang, et al. (2020)	Singapore	Cohort	Dialysate leak	187	Hemodialysis	Mean (SD): Urgent start: 64 (58–72) Conventional start: 61 (51–70)	48.66%	Low
47	Phongphithakchai, et al. (2022)	Thailand	Cohort	The cost-effectiveness of LRKT with those of non-preemptive KT strategies	140	Kidney transplant	Mean (SD): PE LRKT: 42.9 (15.0) NPE LRKT: 41.9 (15.4) NPE DDKT: 46.4 (9.6)	57.14%	Low
48	Premasathian, et al. (2010)	Thailand	Cohort	To investigate the risk factors for DGF and its outcomes among CD-KT among the Thai population	756	Kidney transplant	Mean (SD): 43.0 (12.6)	62.30%	Low
49	Rashid, et al. (2021)	Malaysia	Cohort	Catheter-related infections in PD patients, which include peritonitis, ESI, and TTI infection rates	126	Peritoneal dialysis	Mean (SD): Coiled PD catheter: 49.9 (16.79) Straight PD catheter: 53.4 (14.67)	54.76%	Moderate
50	Rudiansyah, et al. (2021)	Indonesia	Cross-sectional	To determine the correlation between serum iron levels and transferrin saturation with RET-He in patients with CKD-5D	97	Hemodialysis	Mean (SD): 48 (13)	54.64%	Moderate
51	Saengpanit, et al. (2017)	Thailand	RCT	The mean differences between the 3-month and 6-month CAVI values of the treatment and control groups were assessed by using a general linear model with repeated measures, using the baseline CAVI as the covariate	50	Hemodialysis	Mean (SD): Control: 54.4 (10.7) Treatment: 50.4 (9.5)	56.00%	Low
52	Sangadji, et al. (2020)	Indonesia	Cross-sectional	To determine the relationship of biofilms and blood culture	33	Hemodialysis	None	None	Moderate
53	Santika, et al. (2021)	Indonesia	Cross-sectional	To determine the factors causing chronic kidney disease in patients undergoing hemodialysis therapy	307	Hemodialysis	None	70.36%	High
54	Segasothy, et al. (1986)	Malaysia	Cross-sectional	To determine the pattern of analgesic consumption in patients with ESRD requiring dialysis and transplantation in Malaysia	180	Renal replacement therapy	None	None	Moderate
55	Seng, et al. (2018)	Singapore	Cohort	To examine the prevalence of hypercalcemia as well as to identify risk factors associated with hypercalcemia in pre-dialysis patients	557	CKD4, 5ND	Median (range): 73 (63–81)	47.40%	Low
56	Shahar, et al. (2021)	Malaysia	Cross-sectional	To evaluate the prevalence of CRBSI and CC among HD patients	175	Hemodialysis	Median (range): CRBSI: 66 (59–72) CC: 64 (55–73)	59.43%	Moderate
57	Siagian, et al. (2018)	Indonesia	Cross-sectional	To identify the cause of chronic renal disease at the age of under 45 years old in hemodialysis unit	212	Hemodialysis	None	57.55%	Moderate
58	Suandewi, et al. (2020)	Indonesia	Cross-sectional	To determine the sociodemographic and clinical profile of patients with CKD stage 5 who underwent regular hemodialysis	77	Hemodialysis	None	63.64%	Moderate
59	Supasyndh, et al. (2009)	Thailand	Cross-sectional	To compare the nutrition status of patients on twice-weekly HD with patients on thrice-weekly HD by using simple several methods	144	Hemodialysis	Mean (SD): Thrice weekly HD: 47.78 (9.89) Twice weekly HD: 41.63 (10.47)	50.00%	Low
60	Supit, et al. (2019)	Indonesia	Cross-sectional	To provide the latest update on the number and demographics of kidney transplant in Indonesia	203	Kidney transplant	Median (range): 35.4 (15–57)	65.52%	High
61	Surendra, et al. (2019)	Malaysia	Cross-sectional	To estimate health utilities, to compare the HRQOL of HD and CAPD patients, and to identify factors associated with HRQOL	141	CKD 5D	Mean (SD): 53.7 (14.2)	55.32%	Low
62	Suwan. (2011)	Thailand	Cross-sectional	To examine the current status of parathyroid disease, especially hyperparathyroidism	173	Peritoneal dialysis	Mean (SD): 52.43 (14.0)	47.98%	Low
63	Tan. (2014)	Brunei Darussalam	Registry	To compare KPI recorded in the BDTR and department records against international practice	512	ESKD on RRT	Median: 53	50.98%	Not assessed
64	Tan, et al. (2014)	Brunei Darussalam	Cross-sectional	To review renal transplant practice and trend	49	Kidney transplant	Median: 31	65.31%	Moderate
65	Tan, et al. (2019)	Singapore	Cross-sectional	To quantify levels of decision regret among a sample of dialysis patients currently on treatment	103	Hemodialysis and peritoneal dialysis	Mean (SD): 78.2 (5.0)	51.46%	Low
66	Tan, et al. (2020)	Singapore	Cohort	To report the immediate and patency outcomes of arteriovenous fistula (AVF) and arteriovenous graft (AVG) after endovascular thrombectomy	294	Hemodialysis	Mean (SD): 63.7 (11.7)	48.64%	Moderate
67	Tang, et al. (2019)	Malaysia	Cohort	To determine the demographic characteristics of our local patient population who are receiving continuous ambulatory peritoneal dialysis (CAPD) as well as to study the incidence, the microbiological infections and the outcome of CAPD peritonitis	57	Peritoneal dialysis	Mean (SD): 45.5 (15.1)	45.61%	Moderate
68	Teo, et al. (2019)	Singapore	Cohort	To determine whether the spectrum of malignancies has changed in the current era of immunosuppression	489	Kidney transplant	Median (range): Without malignancy: 44.5 (15–67) With malignancy: 50 (18–65)	49.90%	Moderate
69	Tng, et al. (2020)	Singapore	Cohort	To validate this failure to maturation (FTM) equation in our local population setting, and if the equation was non-discriminative	694	Hemodialysis	Median (IQR): 64 (17.0)	62.25%	Low
70	Vareesangthip. (2017)	Thailand	Cohort	To describe patient profiles and treatments, to determine coagulative complications during the study period	598	Hemodialysis	Mean (SD): 55.4 (14.7)	55.69%	Moderate
71	Viboon, et al. (2018)	Thailand	Cohort	To determine the prevalence of AT1R-Ab among pre-transplantation Thai patients and to compare the association of patient demographics with AT1R-Ab levels	70	Kidney transplant	Mean (SD): AT1R-Ab + : 33.5 (14.6) AT1R-Ab at risk: 44.7 (13.3) AT1R-Ab-: 46.1 (10.5)	74.29%	Low
72	Wei, et al. (2021)	Malaysia	Cohort	To find out the total number of elderly patients initiated on dialysis from a tertiary center in Sabah during the stated period, the epidemiology and characteristics of the patients, and the preparedness and the survival of the patients	263	Hemodialysis and peritoneal dialysis	Mean (SD): 70.7 (4.343) Range: 65–85	55.13%	Low
73	Wong, et al. (2016)	Malaysia	Cross-sectional	To assess the clinical impact of education on determining advance care planning (ACP) decisions among ESRD patients on regular hemodialysis (HD)	58	Hemodialysis	Mean (SD): 59.5 (10.9)	55.17%	Moderate
74	Wong,et al. (2022)	Malaysia	Cross-sectional	To study the factors associated with MAFLD and advanced liver fibrosis in these patients	447	Hemodialysis	Median (range): 59 (50–67)	55.03%	Low
75	Wongpraparut, et al. (2021)	Thailand	Cohort	One-year clinical success after percutaneous transluminal renal angioplasty (PTRA) versus PTRA with stenting (PTRAS)	65	Kidney transplant	Mean (SD): 42.5 (11.9)	61.54%	Moderate
76	Yap, et al. (2018)	Singapore	Cohort	The complication rates and factors predicting catheter-related bloodstream infections and mortality rates in patients who were initiated on hemodialysis	677	Hemodialysis	Mean (SD): With CRBSI: 58.5 (11.3) With no CRBSI: 57.3 (11.8)	59.68%	Moderate
77	Yusop, et al. (2013)	Malaysia	Cross-sectional	To determine the relationship between medical history, hemodialysis treatment and nutritional status with quality of life in hemodialysis patients	90	Hemodialysis	Mean (SD): 49.7 (14.1)	48.89%	Moderate
78	Zakaria, et al. (2021)	Malaysia	Cross-sectional	To evaluate the use of traditional and complementary medicine (TCM) among chronic haemodialysis patients in Malaysia	329	Hemodialysis	Mean (SD): 54.9 (12.5)	54.71%	Low
79	Zamri, et al. (2021)	Malaysia	Cross-sectional	To investigate the proportion of ESRD patients without ACS but with elevated hs-cTnT levels above the 99th percentile URL and to provide knowledge on the ranges of hs-cTnT values among dialysis-dependent ESRD patients without ACS	150	Hemodialysis and peritoneal dialysis	Mean (SD): 45.19 (16.36)	38.00%	Moderate
80	Zukiman, et al. (2017)	Malaysia	Cross-sectional	Examined the physical symptom burden (prevalence and severity) among dialysis and nondialysis ESRD patients, its correlation with negative emotional states (depression, stress, and anxiety), and reasons underlying patients’ decisions not to undergo dialysis	187	CKD 5ND and 5D	Mean (SD): ND: 60.97 (13.89) D: 57.72 (13.54)	52.41%	Low
81	Zukmin, et al. (2017)	Brunei Darussalam	Cohort	To compare the survival probability, sociodemographic, and clinical characteristics of multidisciplinary predialysis education (MPE) and non- MPE/crashlander patients during the study period	350	Hemodialysis	Median (IQR): 56.0 (18.0)	56.57%	Moderate

AAC abdominal aortic calcification; ACP advance care planning; ACS acute coronary syndrome; ATG antithymocyte globulin; AT1R-Ab anti-angiotensin II type 1-receptor antibodies; AVF arteriovenous fistula; AVG arteriovenous graft; BDTR Brunei Darussalam Transplant Registry; CAPD continuous ambulatory peritoneal dialysis; CAVI cardio-ankle vascular index; CC catheter colonization; CD-KT cadaveric kidney transplantation; CKD chronic kidney disease; CKDND chronic kidney disease-non dialysis; CKD5D chronic kidney disease stage 5-dialysis; CMV cytomegalovirus; CRBSI catheter-related blood stream infections; CYP cytochrome P450; DD deceased donation; DDKT deceased-donation kidney transplantation; DGF delayed graft function; DKT deceased-donation kidney transplantation; EGD esophageal-gastroduodenoscopy; ESI exit-site infections; ESKD end-stage kidney disease; ESRD end-stage renal disease; FTM failure to maturation; HBV hepatitis B virus; HCV hepatitis C virus; HD hemodialysis; HLA Human leukocyte antigens; HRQoL health-related quality of life; hs-cTnT high-sensitive cardiac troponin T; IDWG interdialytic weight gains; IPD incremental peritoneal dialysis; KDQOL-SF Kidney Disease Quality of Life Short Form; KPI key performance indicators; KT kidney transplantation; LD living donation; LKT living-donation kidney transplantation; LRKT living-related kidney transplantation; MAFLD metabolic dysfunction-associated fatty liver disease; MPE multidisciplinary predialysis education; NPE non-pre-emptive; PAC pelvic artery calcification; PE pre-emptive; PEP patient educational program; PTRA percutaneous transluminal renal angioplasty; PTRAS percutaneous transluminal renal angioplasty with stenting; QoL quality of life; RET-He Reticulocyte haemoglobin equivalent; RRT renal replacement therapy; SARS-Cov-2 severe acute respiratory syndrome coronavirus 2; SC subcutaneous; SD standard deviation; TCM traditional and complementary medicine; TTI tunnel tract infections; UGI upper gastrointestinal; URL upper reference limit

### Etiology of Advanced CKD Among South East Asia Countries

The pooled proportions for different underlying renal conditions were: diabetic kidney disease 29.2% (95%CI 23.88–34.78), glomerulonephritis 20.0% (95%CI 16.84–23.38), hypertensive nephrosclerosis 16.8% (95%CI 14.05–19.70), other 8.6% (95%CI 6.97–10.47), unknown 7.5% (95%CI 4.32–11.50), and polycystic kidney disease 0.7% (95%CI 0.40–1.16) (Table 3 and Supplementary Table S4). Heterogeneity between studies was observed in all etiological diagnosis (Supplementary Figure S2-7).

**Table 3:** Etiology of advanced-CKD in Southeast Asia

Etiology	Prevalence (%)	95% CI	*I*^*2*^ test (%)	*p*-value
Diabetic kidney disease	29.2	23.88–34.78	99.12	< 0.001
Hypertensive nephrosclerosis	16.8	14.05–19.70	97.68	< 0.001
Polycystic kidney disease	0.7	0.40–1.16	89.68	< 0.001
Glomerulonephritis	20.0	16.84–23.38	98.04	< 0.001
Other	8.6	6.97–10.47	96.45	< 0.001
Unknown	7.5	4.32–11.50	99.29	< 0.001

Significant heterogeneity between studies is observed (*p* < 0.001 with *I*^*2*^ exceeding 89% for all six-etiology groups)

CI confidence interval

Sensitivity analysis was conducted in 34 studies with low risk of bias, including a total of 12 218 patients, across five countries, namely Indonesia (1 study, n = 389), Malaysia (7 studies, n = 1664), Myanmar (1 study, n = 54), Singapore (9 studies, n = 4450), and Thailand (16 studies, n = 5661) (Table 4, Supplementary Figure S8-13).Overall, the results are similar to the main analysis. It is evident that the most common cause of CKD is still diabetic kidney disease (29.9%, 95%CI 21.97–38.41), followed by glomerulonephritis (19.4%, 95%CI 14.73–24.49), and hypertensive nephrosclerosis (13.4%, 95%CI 9.34–17.94). However, often the underlying disease was unknown (13.6%, 95%CI 6.82–22.11), possibly due to the majority of studies being from Thailand, where a high rate of unknown causes has been reported. Additionally, the prevalence of polycystic kidney disease remains low compared to the prevalence before sensitivity analysis (0.9% vs. 0.7%). Nevertheless, there is a significant heterogeneity observed between the studies after this analysis.

**Table 4:** Sensitivity analysis on the etiology of advanced-CKD in Southeast Asia

Etiology	Prevalence (%)	95% CI	*I*^*2*^ test (%)	*p*-value
Diabetic kidney disease	29.9	21.97–38.41	98.96	< 0.001
Hypertensive nephrosclerosis	13.4	9.34–17.94	97.84	< 0.001
Polycystic kidney disease	0.9	0.38–1.74	90.26	< 0.001
Glomerulonephritis	19.4	14.73–24.49	97.75	< 0.001
Others	6.6	4.27–9.38	96.60	< 0.001
Unknown	13.6	6.82–22.11	99.33	< 0.001

Significant heterogeneity between studies is observed (*p* < 0.001 with *I*^*2*^ exceeding 90% for all six-etiology groups)

CI confidence interval

### Primary Renal Disease Across Countries

Results were stratified to determine the proportion of underlying kidney disease in 9 countries. The analysis revealed that diabetic kidney disease is most commonly found in Malaysia (40.3%, 95%CI 28.78–52.28), Singapore (39.2%, 95%CI 28.12–50.82), and Brunei Darussalam (37.9%, 95%CI 21.90–55.38) (Table 5). On the other hand, hypertensive nephrosclerosis is the main cause of kidney disease in Myanmar (53.7%, 95%CI 40.61–66.31), Indonesia (48.7%, 95%CI 37.73–59.77), and Cambodia (46.7%, 95%CI 41.89–51.54). Glomerulonephritis was found to be the primary cause in Vietnam (57.3%, 95%CI 51.74–62.80) and the Philippines (42.2%, 95%CI 18.70–67.59). Although the overall proportion of unknown causes is less than 10%, it remains prevalent in countries like Malaysia and Thailand (10.4%, 95%CI 5.24–17.04 and 23.6%, 95%CI 15.24 –33.04; respectively). Surprisingly, the prevalence of polycystic kidney disease is high in Vietnam (3.5%, 95%CI 1.66–5.94), with smaller percentages reported in Brunei Darussalam and Singapore (1.09%, 95% CI 0.00–4.46 and 1.95%, 95%CI 0.84–3.44; respectively), and remains low (prevalence less than one percent) in the other six countries (Table 5, Supplementary Figure S14-19).

**Table 5:** Stratification of the distribution of primary kidney diseases across countries

Country	Number of studies	Study size	Diabetic kidney disease		Hypertensive nephrosclerosis		Polycystic kidney disease		Glomerulonephritis		Others		Unknown	
			%	95%CI	%	95%CI	%	95%CI	%	95%CI	%	95%CI	%	95%CI
Malaysia	19	3378	**40.3**	28.78–52.28	12.8	8.72–17.47	0.4	0.03–1.08	11.4	6.80–16.93	11.1	7.24–15.56	10.4	5.24–17.04
Indonesia	11	1805	23.2	18.64–28.07	**48.7**	37.73–59.77	0.3	0.00–1.00	9.8	4.41–16.9	11.6	6.50–17.89	0.2	0.00–1.27
Thailand	21	12215	17.9	12.90–23.67	12.3	8.47–16.59	0.3	0.01–0.87	23.2	19.09–27.56	7.0	4.24–10.33	**23.6**	15.24–33.04
Singapore	18	13013	**39.2**	28.12–50.82	8.9	6.63–11.36	1.9	0.84–3.44	30.8	22.46–39.74	5.5	3.28–8.11	2.2	0.38–5.27
Brunei	5	1105	**37.9**	21.90–55.38	20.7	14.80–27.29	1.1	0.00–4.46	14.8	3.11–32.54	7.6	4.27–11.66	9.9	1.86–22.97
Philippine	3	548	21.7	5.89–43.42	14.1	4.50–27.36	0.0	0.00–2.04	**42.2**	18.70–67.59	12.5	0.00–49.90	0.0	0.00–0.02
Vietnam	2	309	12.7	9.16–16.66	16.4	12.41–20.74	3.5	1.66–5.94	**57.3**	51.74–62.80	6.2	3.75–9.28	0.5	0.00–1.70
Myanmar	1	54	12.9	6.42–24.42	**53.7**	40.61–66.31	0.0	0.00–6.64	7.4	2.92–17.55	16.7	9.02–28.74	9.3	4.02–19.91
Cambodia	1	407	8.1	5.83–11.17	**46.7**	41.89–51.54	0.0	0.00–0.94	12.0	9.23–15.56	33.2	28.77–37.88	0.0	0.00–0.94
Overall	81	32834	29.2	23.88–34.78	16.8	14.05–19.70	0.74	0.40–1.16	20.0	16.84–23.38	8.6	6.97–10.47	7.5	4.32–11.50

The texts highlighted in bold indicate the highest prevalence within the countries that were studied

CI confidence interval

### Primary Kidney Diseases Before and After 2000

Further analysis was conducted to examine the occurrence of underlying kidney disease during two different time periods, specifically before and after 2000. Prior to 2000, three reports were completed from Singapore, Thailand, and Malaysia, while the majority of studies (96.3%, n = 78/81 [n = 30 427 subjects]) were carried out after 2000. The most common primary renal disease found before 2000 was unknown causes (35%, 95%CI 19.67–52.04), followed by glomerulonephritis (34.7%, 95%CI 14.97–57.70). After 2000, the majority of diagnoses were related to diabetic kidney disease (30%, 95%CI 24.59–35.97), although glomerulonephritis still accounted for a significant proportion of the study population (19.5%; 95%CI 16.23–22.93). During the two study periods, there was a notable increase in both hypertensive nephrosclerosis and diabetic kidney disease (from 4.9%, 95%CI 0.00–22.78 to 17.4%, 95%CI 14.56–20.41 and from 9.2%, 95%CI 0.00–33.01 to 30%, 95%CI 24.59–35.97, respectively). The prevalence of polycystic kidney disease and other etiology remained stable. In contrast, the occurrence of glomerulonephritis and unknown causes declined (from 34.7%, 95%CI 14.97–57.70 to 19.5%, 95%CI 16.23–22.93, and from 35%, 95%CI 19.67–52.04 to 6.8%, 95%CI 3.64–10.78, respectively) (Table 6, Supplementary Figure S20-25).

**Table 6:** Stratification of the distribution of primary kidney diseases before and after 2000

Study period	Number of studies	Study size	Diabetic kidney disease		Hypertensive nephrosclerosis		Polycystic kidney disease		Glomerulonephritis		Others		Unknown	
			%	95%CI	%	95%CI	%	95%CI	%	95%CI	%	95%CI	%	95%CI
Before 2000	3	2407	9.2	0.00–33.01	4.9	0.00–22.78	0.9	0.00–4.06	34.7	14.97–57.70	8.0	1.87–17.78	**35**	19.67–52.04
After 2000	78	30427	**30.1**	24.59–35.97	17.3	14.56–20.41	0.7	0.39–1.18	19.5	16.23–22.93	8.7	6.93–10.59	6.8	3.64–10.78
Overall	81	32834	29.2	23.88–34.78	16.8	14.05–19.70	0.7	0.40–1.16	20.0	16.84–23.38	8.6	6.97–10.47	7.5	4.32–11.50

The texts highlighted in bold indicate the highest prevalence within the periods that were studied

CI confidence interval

### Primary Renal Disease Across Countries’ Economic Status

The prevalence of primary renal diseases was further investigated based on the economic standing of the country. Most of the studies come from the upper-middle-income countries (51 studies; n = 17,398), while only 7 studies (n = 1,318) come from low-middle-income countries. Among upper-middle-income and high-income countries, diabetic kidney disease is the most common condition (26.8%, 95%CI 21.42–32.60 and 38.8%, 95%CI 29.33–48.79, respectively). On the other hand, glomerulonephritis is more prevalent in lower middle-income countries (33.8%, 95%CI 15.62–54.81), when compared to upper middle-income countries (15.5%, 95%CI 12.53–18.74), but it is also common in high-income nations (27%, 95%CI 19.56–35.22). Lower-middle-income countries report a relatively higher occurrence of hypertensive nephrosclerosis and other etiologies compared to upper-middle- and high-income countries. Additionally, polycystic kidney disease is reported more frequently in high-income countries compared to countries with lower income status (Table 7).

**Table 7:** Stratification of the distribution of primary kidney diseases across economic status

Country’s economic status	Number of studies	Study size	Diabetic kidney disease		Hypertensive nephrosclerosis		Polycystic kidney disease		Glomerulonephritis		Others		Unknown	
			%	95%CI	%	95%CI	%	95%CI	%	95%CI	%	95%CI	%	95%CI
Lower-middle-income	7	1318	16.2	9.01–24.81	24.7	11.16–41.40	0.6	0.00–2.38	**33.8**	15.62–54.81	14	3.90–28.57	0.3	0.00–1.82
Upper-middle-income	51	17398	**26.8**	21.42–32.60	18.8	14.68–23.29	0.4	0.10–0.71	15.5	12.53–18.74	9.34	7.23–11.77	11.5	6.68–17.34
High-income	23	14118	**38.8**	29.33–48.79	10.8	8.36–13.58	1.8	0.80–3.02	27.0	19.56–35.22	5.9	3.89–8.19	3.4	1.19–6.48
Overall	81	32834	29.2	23.88–34.78	16.8	14.05–19.70	0.7	0.40–1.16	20.0	16.84–23.38	8.6	6.97–10.47	7.5	4.32–11.50

The texts highlighted in bold indicate the highest prevalence within the country’s economic status

CI confidence interval

## Discussion

In this meta-analysis, we summarized the evidence using the best available data to estimate the prevalence of the different causes of CKD in Southeast Asian countries. Our results suggest that in the advanced-CKD population and those receiving kidney replacement therapy, diabetic kidney disease is the most prevalent etiology, followed by glomerulonephritis and hypertensive nephrosclerosis. The prevalence of polycystic kidney disease is relatively low. Diabetic kidney disease appears to have the highest prevalence in upper-middle and high-income countries and to have sharply increased after the year 2000. Nevertheless, the proportion of glomerulonephritis should not be disregarded.

Our study aimed to examine the variation in CKD etiology before and after the year 2000 in Southeast Asian countries, as well as among different socioeconomic groups [16]. Global trends indicate that non-communicable diseases have become more prevalent since the turn of the century, resulting in increased mortality rates [17]. Diabetes is a major contributor to this trend, with its increasing prevalence mirroring the rise in CKD prevalence. Furthermore, the longer life expectancies observed after the year 2000 increase the likelihood of developing chronic diseases [17]. Likewise, advancements in health have been linked to economic growth through improvement in nutrition, enhancements to public health infrastructure like water purity and sanitation, and more effective medical treatments like antibiotics and vaccinations [18]. Subsequently, this situation is believed to impact the underlying kidney disease across various time periods and countries with differing levels of wealth.

In the entire cohort, it is evident that diabetes mellitus is the leading cause of primary kidney disease among studies. This trend is particularly observed in upper-middle and high-income countries. And a significant increase in the prevalence of diabetic kidney disease is observed after the year 2000. This observation resembles the trend in western countries like Europe and the US. The incidence of diabetic kidney disease in kidney failure patients receiving KRT is reported to be 23% (32.4 per million population) in Europe in 2021, up to 46.8% in the US in 2019 [19–21]. Diabetes mellitus is a non-communicable disease that has a major impact on global health, particularly in relation to kidney disease. Based on data from the Global Burden of Disease Study 2019 (GDB 2019), approximately two thirds of DALYs in 2019 from known causes were attributable to CKD caused by type 2 diabetes mellitus (T2DM) and hypertension, which also showed the largest increase in the age-standardized DALY rate [1, 22]. Between 2021 and 2045, it is expected that the global rate of adults living with T2DM would escalate by 46%; where Southeast Asia will provide the largest increase (68%), following Africa and the Middle East & North Africa [23, 24]. A study involving 10 Asian countries (China, Hong Kong, Indonesia, Malaysia, Pakistan, Philippines, Singapore, South Korea, Taiwan and Thailand) revealed that a significant proportion of hypertensive type 2 diabetic patients (58.6%) exhibit either micro or macroalbuminuria, which could lead to kidney complications [25]. Moreover, the epidemic of metabolic syndrome [26], is the primary cause of the rising burden of T2DM. This increase in T2DM has been linked to a rise in processed food consumption, a decline in physical activity, and an increase in sedentary behavior [26]. The so-called western lifestyle is associated with increased urbanization and industrial advancement on a worldwide scale. The prevalence of metabolic syndrome among Asian populations was reported to be about 10–30%, resembling that among Western countries [26–30]. Moreover, there is evidence linking the hazard of metabolic syndrome to an increased risk of developing CKD. Additionally, it is estimated that 20–50% of T2DM patients will eventually develop DKD [31–33]. The rapid growth of the ageing population in Asia has also altered the disease epidemiology. From this standpoint, we strongly support the international recommendation to early identify the development of CKD in the high-risk population (such as those with T2DM) in Southeast Asia [34].

Our research has demonstrated that glomerulonephritis is prevalent in a similar manner to earlier findings from Southeast Asia [35, 36]. This finding remains consistent even after conducting sensitivity analysis and when comparing different countries, study periods, and income levels. However, due to the significant heterogeneity in the studies, we should interpret this result with caution. For instance, the prevalence of glomerulonephritis is particularly high in Vietnam and the Philippines (over 40%), while it consistently stays around 10% in other countries, except for Myanmar and Indonesia (less than 10%). Similarly, there are notable variations in the occurrence rate of unknown causes across nations. This difference may be attributed to factors beyond sample size and demographics, such as variations in healthcare infrastructure, availability of diagnostic equipment for kidney biopsies, as well as the number of kidney specialists and pathologists. Nevertheless, it is important to acknowledge that Southeast Asia has distinct characteristics compared to other regions worldwide, including diverse economic levels that can influence healthcare systems. The ongoing prevalence of infectious diseases (such as Malaria, Leptospirosis, Dengue, Hepatitis, HIV etc.) and the potential risk of kidney diseases caused by traditional medicines or environmental toxins should also be considered. IgA nephropathy has been identified as a major cause of glomerulonephritis in Asia [37–39]. This study presents a relatively high rate of glomerulonephritis, which may reflect the actual estimate of the disease prevalence in the population due to the aforementioned reasons. Additionally, the under-reporting of glomerulonephritis incidence, particularly in cases where etiological diagnosis cannot be performed due to late-stage chronic kidney disease or inadequate healthcare facilities, should also be taken into account. In contrast to the international recommendations that suggest performing urine albumin to creatinine ratio (uACR) tests in high-risk individuals, these findings emphasize the importance of identifying this specific population group and conducting simple urinalysis tests to detect not only proteinuria but also hematuria or abnormal sediment/cast associated with glomerular diseases.

Another interesting observation is the consistently low prevalence of autosomal dominant polycystic kidney disease (ADPKD) in this cohort, although a higher prevalence is observed among high-income countries. Even comparing to patients receiving KRT, the prevalence of ADPKD in our cohort was much lower than in the Europe (9%) and in the US (9.5% in dialysis and 15.7% in kidney transplant population) [40]. Autosomal dominant polycystic kidney disease affects all racial groups worldwide and is the most common monogenetic disorder of the kidney [41]. The lower prevalence observed in our study may be attributed to under-reporting, which could be due to the inability to perform etiological diagnosis, especially in lower-middle-income countries with limited healthcare facilities. In addition, it might reflect the limited access to nephrology or dialysis facilities.

This meta-analysis included studies with unidentified sources of bias and it was challenging to measure these publication bias. Nonetheless, we made several attempts to reduce the possibility of bias. Studies with a wide range of publication dates were taken into account in this meta-analysis, which also included articles that were published in both English and Bahasa Indonesia languages. Two reviewers individually looked into each study selection procedure. The pooled prevalence from sensitivity analysis was estimated from studies with moderate to high quality.

To our best knowledge, this study is the first to identify the underlying kidney disease distribution in the advanced-CKD population of Southeast Asia by conducting an extensive search across multiple databases and including a large sample size. The manuscript makes a valuable contribution to understanding CKD etiologies in Southeast Asia. However, it is important to acknowledge certain limitations: we included observational, investigational, and registry studies without considering their methodology; not all Southeast Asian countries were included in the study; and the establishment of etiological diagnosis was not clearly defined in each study, which may have led to incorrect classification of some causes. Moreover, the small number of study participants in certain countries making comparisons between countries less reliable. Finally, we acknowledge that relevant studies could be excluded due to language restrictions and there were possible language bias exist.

In conclusion, our meta-analysis indicates that in the advanced-CKD population and patients receiving kidney replacement therapy in Southeast Asia, diabetic kidney disease is the most prevalent etiology, followed by glomerulonephritis and hypertensive nephrosclerosis. The prevalence of diabetic kidney disease is highest in upper-middle-income and high-income countries and has significantly increased after 2000. To reduce the burden of the disease, it is essential to implement an effective screening program for high-risk populations (those with diabetes mellitus). This program should consider building infrastructure, improving access to diagnostic tests for early detection of CKD, and increasing public awareness about kidney health and risk factors.

## Supplementary Information

Below is the link to the electronic supplementary material.

Supplementary file1 (DOCX 14125 KB)

## Data Availability

All data generated and analyzed during this study are included in the main manuscript or supplementary files.
