# Supplementary material for: The Etiology of Advanced Chronic Kidney Disease in Southeast Asia: A Meta-analysis
Source: J Epidemiol Glob Health. 2024 Apr 8;14(3):740–64. doi: 10.1007/s44197-024-00209-5 (PMC11442843; doi:10.1007/s44197-024-00209-5)
Supplement: Supplementary file 1 — Supplementary file1 (DOCX 14125 KB) [file 44197_2024_209_MOESM1_ESM.docx]

Supplementary Form S1. MOOSE (Meta-analyses Of Observational Studies in Epidemiology) Checklist

| **Reporting Criteria** | **Reported (Yes/No)** | | | **Reported on Page No.** | | |
| --- | --- | --- | --- | --- | --- | --- |
| **Reporting of Background** |  | | |  | | |
| Problem definition |  | Yes |  |  | 4 |  |
| Hypothesis statement |  | Yes |  |  | 4 |  |
| Description of Study Outcome(s) |  | Yes |  |  | 7 |  |
| Type of exposure or intervention used |  | No |  |  |  |  |
| Type of study design used |  | Yes |  |  | 6 |  |
| Study population |  | Yes |  |  | 6 |  |
| **Reporting of Search Strategy** |  | | |  | | |
| Qualifications of searchers (eg, librarians  and investigators) | Yes | | | 7 | | |
| Search strategy, including time period  included in the synthesis and keywords | Yes | | | 6 | | |
| Effort to include all available studies,  including contact with authors | Yes | | |  | | |
|  |  |  |  |  | 6 |  |
| Databases and registries searched |  | Yes |  |  | 6 |  |
| Search software used, name and version, including special features used  (eg, explosion) | No | | |  | | |
| Use of hand searching (eg, reference  lists of obtained articles) | No | | |  | | |
| List of citations located and those  excluded, including justification | Yes | | | Table S1 | | |
| Method for addressing articles  published in languages other than English | Yes | | | 6 | | |
| Method of handling abstracts and  unpublished studies | Yes | | | 6 | | |
| Description of any contact with authors |  | No |  |  |  |  |
| **Reporting of Methods** |  | | |  | | |
| Description of relevance or appropriateness of studies assembled for  assessing the hypothesis to be tested | Yes | | | 9 | | |
| Rationale for the selection and coding of data (eg, sound clinical principles or  convenience) | Yes | | | 7 | | |
| Documentation of how data were classified and coded (eg, multiple raters,  blinding, and interrater reliability) | Yes | | | 7 | | |
| Assessment of confounding (eg, comparability of cases and controls in  studies where appropriate | No | | |  | | |

| **Reporting Criteria** | **Reported (Yes/No)** | | | **Reported on Page No.** | | |
| --- | --- | --- | --- | --- | --- | --- |
| Assessment of study quality, including blinding of quality assessors; stratification or regression on possible  predictors of study results | Yes | | | 7 | | |
| Assessment of heterogeneity |  | Yes |  |  | 8 |  |
| Description of statistical methods (eg, complete description of fixed or random effects models, justification of whether the chosen models account for predictors of study results, dose-response models, or cumulative meta-analysis) in sufficient  detail to be replicated | Yes | | | 8 | | |
| Provision of appropriate tables and  graphics | Yes | | | 20-32 | | |
| **Reporting of Results** |  | | |  | | |
| Table giving descriptive information for  each study included | Yes | | | 22-29 | | |
| Results of sensitivity testing (eg,  subgroup analysis) | Yes | | | 30 | | |
| Indication of statistical uncertainty of  findings | No | | |  | | |
| **Reporting of Discussion** |  | | |  | | |
| Quantitative assessment of bias (eg,  publication bias) | Yes | | | 14 | | |
| Justification for exclusion (eg, exclusion  of non–English-language citations) | Yes | | | 15 | | |
| Assessment of quality of included studies |  | Yes |  |  | 14-15 |  |
| **Reporting of Conclusions** |  | | |  | | |
| Consideration of alternative explanations  for observed results | Yes | | | 14-15 | | |
| Generalization of the conclusions (ie, appropriate for the data presented and  within the domain of the literature review) | Yes | | | 15 | | |
| Guidelines for future research |  | Yes |  |  | 15 |  |
| Disclosure of funding source |  | Yes |  |  | 15 |  |

**Supplementary Table S1. Search strategy and selection criteria**

| **Database** | **Date of search** | **MeSH terms** | **Results** |
| --- | --- | --- | --- |
| **PubMed [http://www.ncbi.nlm.nih.gov/entrez/query.fcgi?otool=leiden](http://www.ncbi.nlm.nih.gov/entrez/query.fcgi?otool=leiden)** | 01-09-2022 | ("Renal Insufficiency, Chronic"[Mesh] OR "chronic renal insufficienc*"[tw] OR "chronic renal disease*"[tw] OR "chronic kidney disease*"[tw] OR "chronic renal failure*"[tw] OR "chronic kidney failure*"[tw] OR "CKD"[tiab] OR "CRF"[tiab]) | 190953 |
|  |  | ("Asia, Southeastern"[Mesh] OR "South East Asia*"[tw] OR "Southeast Asia*"[tw] OR "South Eastern Asia*"[tw] OR "Southeastern Asia*"[tw] OR "Indonesia*"[tw] OR "Java*"[tw] OR "Malaysia*"[tw] OR "Thai*"[tw] OR "Singapore*"[tw] OR "Vietnam*"[tw] OR "Cambodia*"[tw] OR "Kampuch*"[tw] OR "Timor"[tw] OR "Philippine*"[tw] OR "Brunei*"[tw] OR "Myanmar*"[tw] OR "Burma"[tw] OR "Burmese"[tw] OR "Laos"[tw] OR "Laotian"[tw]) | 183077 |
|  |  | ("Renal Insufficiency, Chronic"[Mesh] OR "chronic renal insufficienc*"[tw] OR "chronic renal disease*"[tw] OR "chronic kidney disease*"[tw] OR "chronic renal failure*"[tw] OR "chronic kidney failure*"[tw] OR "CKD"[tiab] OR "CRF"[tiab]) AND ("Asia, Southeastern"[Mesh] OR "South East Asia*"[tw] OR "Southeast Asia*"[tw] OR "South Eastern Asia*"[tw] OR "Southeastern Asia*"[tw] OR "Indonesia*"[tw] OR "Java*"[tw] OR "Malaysia*"[tw] OR "Thai*"[tw] OR "Singapore*"[tw] OR "Vietnam*"[tw] OR "Cambodia*"[tw] OR "Kampuch*"[tw] OR "Timor"[tw] OR "Philippine*"[tw] OR "Brunei*"[tw] OR "Myanmar*"[tw] OR "Burma"[tw] OR "Burmese"[tw] OR "Laos"[tw] OR "Laotian"[tw]) | 1220 |
|  |  | ("Renal Insufficiency, Chronic"[Mesh] OR "chronic renal insufficienc*"[ti] OR "chronic renal disease*"[ti] OR "chronic kidney disease*"[ti] OR "chronic renal failure*"[ti] OR "chronic kidney failure*"[ti] OR "CKD"[ti] OR "CRF"[ti] OR "Renal Dialysis"[Mesh] OR "Dialysis"[ti] OR "Dialytic"[ti] OR "Hemodialy*"[ti] OR "Haemodialy*"[ti]) AND ("Asia, Southeastern"[Mesh] OR "South East Asia*"[tw] OR "Southeast Asia*"[tw] OR "South Eastern Asia*"[tw] OR "Southeastern Asia*"[tw] OR "Indonesia*"[tw] OR "Java*"[tw] OR "Malaysia*"[tw] OR "Thai*"[tw] OR "Singapore*"[tw] OR "Vietnam*"[tw] OR "Cambodia*"[tw] OR "Kampuch*"[tw] OR "Timor"[tw] OR "Philippine*"[tw] OR "Brunei*"[tw] OR "Myanmar*"[tw] OR "Burma"[tw] OR "Burmese"[tw] OR "Laos"[tw] OR "Laotian"[tw]) AND ("endstage"[tw] OR "stage*"[tw] OR "Staging"[tw]) | 451 |
|  |  | ("Renal Insufficiency, Chronic"[Mesh] OR "chronic renal insufficienc*"[ti] OR "chronic renal disease*"[ti] OR "chronic kidney disease*"[ti] OR "chronic renal failure*"[ti] OR "chronic kidney failure*"[ti] OR "CKD"[ti] OR "CRF"[ti] OR "Renal Dialysis"[Mesh] OR "Dialysis"[ti] OR "Dialytic"[ti] OR "Hemodialy*"[ti] OR "Haemodialy*"[ti] OR "Kidney Transplantation"[Mesh] OR "Kidney Transplant*"[ti] OR "renal Transplant*"[ti]) AND ("Asia, Southeastern"[Mesh] OR "South East Asia*"[tw] OR "Southeast Asia*"[tw] OR "South Eastern Asia*"[tw] OR "Southeastern Asia*"[tw] OR "Indonesia*"[tw] OR "Java*"[tw] OR "Malaysia*"[tw] OR "Thai*"[tw] OR "Singapore*"[tw] OR "Vietnam*"[tw] OR "Cambodia*"[tw] OR "Kampuch*"[tw] OR "Timor"[tw] OR "Philippine*"[tw] OR "Brunei*"[tw] OR "Myanmar*"[tw] OR "Burma"[tw] OR "Burmese"[tw] OR "Laos"[tw] OR "Laotian"[tw]) AND ("endstage"[tw] OR "stage*"[tw] OR "Staging"[tw]) | 483 |
| **Embase http://ovidsp.ovid.com/ovidweb.cgi?T=JS&PAGE=main&MODE=ovid&D=oemezd** | 01-09-2022 | ( exp chronic kidney failure/ OR "chronic renal insufficienc*".ti. OR "chronic renal disease*".ti. OR "chronic kidney disease*".ti. OR "chronic renal failure*".ti. OR "chronic kidney failure*".ti. OR "CKD".ti. OR "CRF".ti. OR exp hemodialysis/ OR "Dialysis".ti. OR "Dialytic".ti. OR "Hemodialy*".ti. OR "Haemodialy*".ti. OR exp kidney transplantation/ OR "Kidney Transplant*".ti. OR "renal Transplant*".ti.) AND (exp Southeast Asia/ OR "South East Asia*".mp. OR "Southeast Asia*".mp. OR "South Eastern Asia*".mp. OR "Southeastern Asia*".mp. OR "Indonesia*".mp. OR "Java*".mp. OR "Malaysia*".mp. OR "Thai*".mp. OR "Singapore*".mp. OR "Vietnam*".mp. OR "Cambodia*".mp. OR "Kampuch*".mp. OR "Timor".mp. OR "Philippine*".mp. OR "Brunei*".mp. OR "Myanmar*".mp. OR "Burma".mp. OR "Burmese".mp. OR "Laos".mp. OR "Laotian".mp.) AND (exp staging/ OR "endstage".mp. OR "stage*".mp. OR "Staging".mp.) | 966 |
|  |  | ( exp chronic kidney failure/ OR "chronic renal insufficienc*".ti. OR "chronic renal disease*".ti. OR "chronic kidney disease*".ti. OR "chronic renal failure*".ti. OR "chronic kidney failure*".ti. OR "CKD".ti. OR "CRF".ti. OR exp hemodialysis/ OR "Dialysis".ti. OR "Dialytic".ti. OR "Hemodialy*".ti. OR "Haemodialy*".ti. OR exp kidney transplantation/ OR "Kidney Transplant*".ti. OR "renal Transplant*".ti.) AND (exp Southeast Asia/ OR "South East Asia*".mp. OR "Southeast Asia*".mp. OR "South Eastern Asia*".mp. OR "Southeastern Asia*".mp. OR "Indonesia*".mp. OR "Java*".mp. OR "Malaysia*".mp. OR "Thai*".mp. OR "Singapore*".mp. OR "Vietnam*".mp. OR "Cambodia*".mp. OR "Kampuch*".mp. OR "Timor".mp. OR "Philippine*".mp. OR "Brunei*".mp. OR "Myanmar*".mp. OR "Burma".mp. OR "Burmese".mp. OR "Laos".mp. OR "Laotian".mp.) AND (exp staging/ OR "endstage".mp. OR "stage*".mp. OR "Staging".mp.) NOT (conference OR conference abstract OR "conference review").pt. | 534 |
| **Cochrane** [**https://www.cochranelibrary.com/view/0/index.html**](https://www.cochranelibrary.com/view/0/index.html) | 01-09-2022 | (chronic kidney failure OR "chronic renal insufficienc*" OR "chronic renal disease*" OR "chronic kidney disease*" OR "chronic renal failure*" OR "chronic kidney failure*" OR "CKD" OR "CRF" OR hemodialysis OR "Dialysis" OR "Dialytic" OR "Hemodialy*" OR "Haemodialy*" OR kidney transplantation OR "Kidney Transplant*" OR "renal Transplant*"):ti AND (Southeast Asia OR "South East Asia*" OR "Southeast Asia*" OR "South Eastern Asia*" OR "Southeastern Asia*" OR "Indonesia*" OR "Java*" OR "Malaysia*" OR "Thai*" OR "Singapore*" OR "Vietnam*" OR "Cambodia*" OR "Kampuch*" OR "Timor" OR "Philippine*" OR "Brunei*" OR "Myanmar*" OR "Burma" OR "Burmese" OR "Laos" OR "Laotian"):ti,ab,kw AND (staging OR "endstage" OR "stage*" OR "Staging"):ti,ab,kw | 46 |
| **Scopus** | 18-10-2022 | ( ( {chronic renal insufficienc*} OR {chronic renal disease*} OR {chronic kidney disease*} OR {chronic renal failure*} OR {chronic kidney failure*} OR {CKD} OR {CRF} OR {Renal Dialysis} OR {Dialysis} OR {Dialytic} OR {Hemodialy*} OR {Haemodialy*} OR {Kidney Transplant*} OR {renal Transplant*} ) AND ( {South East Asia*} OR {Southeast Asia*} OR {South Eastern Asia*} OR {Southeastern Asia*} OR {Indonesia*} OR {Java*} OR {Malaysia*} OR {Thai*} OR {Singapore*} OR {Vietnam*} OR {Cambodia*} OR {Kampuch*} OR {Timor} OR {Philippine*} OR {Brunei*} OR {Myanmar*} OR {Burma} OR {Burmese} OR {Laos} OR {Laotian} ) AND ( {endstage} OR {stage*} OR {Staging} ) ) | 33 |
| **National kidney registry and national library repositories** | 20-07-2023 | The hand-search was conducted on the national kidney registries to investigate the etiology of advanced-chronic kidney disease. Additionally, we utilized the national repository libraries and in Bahasa Indonesia for accessing Indonesian's national repositories.  National repositories used are as follow:   1. Indonesia:    1. **neliti** (keywords: ëtiology” and “chronic kidney disease” 🡪 10 articles),    2. **repositori** (keywords: ëtiology” and “chronic kidney disease” 🡪 0 article; keywords: ëtiologi”and “penyakit ginjal kronik”🡪 0 article; keywords: ëtiologi”and “penyakit ginjal kronik” and “dialysis”🡪 0 article),    3. **Unair repository** (keywords: ëtiology” and “chronic kidney disease” 🡪 122 articles),    4. **garuda** (keywords: ëtiology” and “chronic kidney disease” 🡪 1 article; keywords: ëtiologi” and “penyakit ginjal kronik” 🡪 0 article). 2. Philippine:    1. **National Library of the Philippine** (keywords: ëtiology” and “chronic kidney disease” 🡪 0 article). 3. Thailand:    1. **Thai National Research Repository** (keywords: ëtiology” and “chronic kidney disease” 🡪 2 article2).   *We were unable to search for other national repository of Laos, Myanmar, Cambodia, East Timor, Vietnam.  **Malaysian Dialysis and Transplant Registry** 🡪 2 articles  **Indonesian Renal Registry** 🡪 1 article  **Thailand Renal Replacement Therapy** 🡪 1 article  **Google scholar**, keywords as follow::   1. "etiology" "dialysis" "chronic kidney disease" "Cambodia" "kidney replacement therapy" 🡪 23 articles 2. "etiology" "dialysis" "chronic kidney disease" "Laos" "kidney replacement therapy" 🡪 8 articles 3. "etiology" "dialysis" "chronic kidney disease" "East Timor" "Timor Leste" "kidney replacement therapy" 🡪 0 articles 4. "etiology" "dialysis" "chronic kidney disease" "Vietnam" "kidney replacement therapy" 🡪 50 articles 5. "etiology" "dialysis" "chronic kidney disease" "Myanmar" "kidney replacement therapy" 🡪 65 articles   **Manual search on other search engine** (keywords: ëtiology” and “chronic kidney disease” 🡪 6 articles).  In total, from hand-search we collected 290 articles:  We exclude 255 articles for any of these reasons:   - Duplication: 6 - Not adult: 21 - Non-SEA: 23 - Not CKD: 74 - Not original investigation: 79 - Not in English or Bahasa: 5 - Not reported etiology of CKD: 37 - Not in human: 7 - Full text not available: 3 | 35 |
| **Inclusion criteria** | - Patients with advanced CKD (stage 4/5), on KRT (hemodialysis, peritoneal dialysis, kidney transplantation) - Study design: randomized controlled trials, observational studies including cohorts, cross-sectional, case-control studies. - Outcomes of interest: reported the etiology/underlying/primary kidney disease. - Adults >15 years | | |
| **Exclusion criteria** | - Patients with acute kidney injury (AKI) - Not original research, including: reviews, letters, editorials, opinion papers, abstracts only, conference abstracts, and study protocols. - Pediatric (<15 years) - Not human - Not in English or Bahasa Indonesia | | |

Initial screening process (n=1131):

excluding duplication (n=277) using EndNote software version 20.2.1.

IRR 1 – at the start of screening (n=854)

Generated coding according to inclusion and exclusion criteria’s.

Three raters individually screened all the studies.

Results were compared and disagreements were resolved through consensus or by involving a third reviewer.

IRR 2 – at the midway through the screening process (n=475)

Revised the coding followed by discussion

81 studies included for final analysis

**Supplementary Figure 1. Flowchart of screening process and the assessment of interrater reliability**

Note: IRR interrater reliability, assessed by intraclass correlation coefficient for three raters.

**Supplementary table S2. Results of Intraclass correlation (ICC) between the three raters**

| **Screening process** | **First IRR** | **Second IRR** |
| --- | --- | --- |
| Total items coded individually: | | |
| n studies (%) | 854 (100) | 475 (100) |
| Intraclass correlation coefficient: | | |
| Average measures | 0.99 | 0.982 |
| 95% Confidence Interval | 0.989 - 0.991 | 0.979 - 0.985 |

Note: ICC estimates and their 95% confident intervals were calculated using SPSS statistical package version 27 (SPSS Inc, Chicago, IL) based on a mean-rating (k = 3), absolute-agreement, 2-way mixed-effects model.

**Supplementary Table S3. Study population**

| **Study population** | **n (%)** |
| --- | --- |
| General population | 822 (3) |
| CKD4, 5ND | 557 (2) |
| CKD pre- and on-dialysis | 458 (1) |
| CKD 5 | 802 (2) |
| CKD 5ND and 5D | 187 (1) |
| CKD 5D (HD and PD) | 6908 (21) |
| HD | 8108 (25) |
| PD | 2517 (8) |
| KT | 9722 (30) |
| HD and KT | 259 (1) |
| RRT | 2494 (8) |
| **Total population** | **32834 (100)** |

**Supplementary Table S4. Prevalence of CKD etiology in South East Asia**

| **No.** | **Author (year)** | **Diabetic kidney disease** | | | | **Hypertension** | | | | **Polycystic kidney disease** | | | | **Glomerulonephritis** | | | | **Others** | | | | **Unknown** | | | |
| --- | --- | --- | --- | --- | --- | --- | --- | --- | --- | --- | --- | --- | --- | --- | --- | --- | --- | --- | --- | --- | --- | --- | --- | --- | --- |
|  |  | **ES** | **95% CI** | | **% Weight** | **ES** | **95% CI** | | **% Weight** | **ES** | **95% CI** | | **% Weight** | **ES** | **95% CI** | | **% Weight** | **ES** | **95% CI** | | **% Weight** | **ES** | **95% CI** | | **% Weight** |
| 1 | Bavanandan, et al. (2015) | 5.50 | 2.55 | 11.49 | 1.23 | 8.26 | 4.40 | 14.95 | 1.22 | 0.00 | 0.00 | 3.40 | 1.15 | 35.78 | 27.41 | 45.12 | 1.23 | 6.42 | 3.15 | 12.67 | 1.21 | 44.04 | 35.08 | 53.40 | 1.23 |
| 2 | Bawazier, et al. (2018) | 15.38 | 7.25 | 29.73 | 1.17 | 43.59 | 29.30 | 59.02 | 1.08 | 0.00 | 0.00 | 8.97 | 0.76 | 12.82 | 5.60 | 26.71 | 1.10 | 28.21 | 16.54 | 43.78 | 1.02 | 0.00 | 0.00 | 8.97 | 1.18 |
| 3 | Bawazier, et al. (2019) | 22.62 | 18.74 | 27.04 | 1.26 | 67.87 | 63.07 | 72.31 | 1.29 | 0.00 | 0.00 | 0.98 | 1.45 | 3.34 | 1.96 | 5.63 | 1.29 | 9.00 | 6.54 | 12.26 | 1.33 | 0.00 | 0.00 | 0.98 | 1.25 |
| 4 | Boongird, et al. (2022) | 18.33 | 10.56 | 29.92 | 1.20 | 18.33 | 10.56 | 29.92 | 1.15 | 0.00 | 0.00 | 6.02 | 0.93 | 21.67 | 13.12 | 33.62 | 1.16 | 41.67 | 30.06 | 54.27 | 1.11 | 0.00 | 0.00 | 6.02 | 1.21 |
| 5 | Bunchorntavakul, et al. (2014) | 4.67 | 2.01 | 10.48 | 1.23 | 0.00 | 0.00 | 3.47 | 1.22 | 5.61 | 2.60 | 11.70 | 1.14 | 24.30 | 17.16 | 33.22 | 1.22 | 6.54 | 3.20 | 12.89 | 1.21 | 58.88 | 49.41 | 67.74 | 1.23 |
| 6 | Bunnag, et al. (2011) | 25.93 | 13.17 | 44.68 | 1.13 | 7.41 | 2.06 | 23.37 | 0.99 | 0.00 | 0.00 | 12.46 | 0.61 | 3.70 | 0.66 | 18.28 | 1.02 | 0.00 | 0.00 | 12.46 | 0.90 | 62.96 | 44.23 | 78.47 | 1.15 |
| 7 | Chan, et al. (2012) | 25.53 | 19.83 | 32.21 | 1.25 | 0.00 | 0.00 | 2.00 | 1.26 | 0.00 | 0.00 | 2.00 | 1.30 | 8.51 | 5.31 | 13.38 | 1.26 | 37.23 | 30.64 | 44.34 | 1.27 | 29.79 | 23.71 | 36.68 | 1.24 |
| 8 | Chan, et al. (2019) | 45.61 | 33.37 | 58.41 | 1.20 | 15.79 | 8.54 | 27.36 | 1.14 | 0.00 | 0.00 | 6.31 | 0.91 | 5.26 | 1.81 | 14.37 | 1.16 | 12.28 | 6.08 | 23.25 | 1.10 | 21.05 | 12.47 | 33.29 | 1.21 |
| 9 | Cheung, et al. (2012) | 65.38 | 54.33 | 74.99 | 1.22 | 24.36 | 16.19 | 34.94 | 1.19 | 0.00 | 0.00 | 4.69 | 1.03 | 3.85 | 1.32 | 10.71 | 1.19 | 6.41 | 2.77 | 14.14 | 1.16 | 0.00 | 0.00 | 4.69 | 1.22 |
| 10 | Chiasakul, et al. (2015) | 11.57 | 7.02 | 18.49 | 1.23 | 4.96 | 2.29 | 10.40 | 1.23 | 2.48 | 0.85 | 7.04 | 1.18 | 37.19 | 29.10 | 46.07 | 1.23 | 4.96 | 2.29 | 10.40 | 1.23 | 38.84 | 30.63 | 47.74 | 1.23 |
| 11 | Chitasombat, et al. (2018) | 10.00 | 3.46 | 25.62 | 1.14 | 0.00 | 0.00 | 11.35 | 1.02 | 3.33 | 0.59 | 16.67 | 0.65 | 30.00 | 16.66 | 47.88 | 1.05 | 10.00 | 3.46 | 25.62 | 0.93 | 53.33 | 36.14 | 69.77 | 1.16 |
| 12 | Chittinandana. (1999) | 28.14 | 26.11 | 30.26 | 1.27 | 16.65 | 15.00 | 18.44 | 1.32 | 0.00 | 0.00 | 0.21 | 1.58 | 20.42 | 18.62 | 22.35 | 1.31 | 12.82 | 11.35 | 14.44 | 1.36 | 21.70 | 19.86 | 23.66 | 1.26 |
| 13 | Chong, et al. (2013) | 34.15 | 26.36 | 42.89 | 1.24 | 23.58 | 16.95 | 31.81 | 1.23 | 5.69 | 2.78 | 11.28 | 1.18 | 21.95 | 15.55 | 30.05 | 1.23 | 10.57 | 6.28 | 17.25 | 1.23 | 14.63 | 9.46 | 21.95 | 1.24 |
| 14 | Chong, et al. (2018) | 30.99 | 21.44 | 42.48 | 1.21 | 35.21 | 25.12 | 46.82 | 1.17 | 7.04 | 3.05 | 15.45 | 0.99 | 16.90 | 9.94 | 27.26 | 1.18 | 9.86 | 4.86 | 18.98 | 1.14 | 8.45 | 3.93 | 17.24 | 1.22 |
| 15 | Choo, et al. (2012) | 58.03 | 54.97 | 61.03 | 1.27 | 15.47 | 13.37 | 17.82 | 1.31 | 1.77 | 1.12 | 2.79 | 1.55 | 23.25 | 20.76 | 25.95 | 1.30 | 1.48 | 0.90 | 2.42 | 1.35 | 0.00 | 0.00 | 0.38 | 1.26 |
| 16 | Choong, et al. (1991) | 2.12 | 1.12 | 3.98 | 1.26 | 0.00 | 0.00 | 0.90 | 1.30 | 1.88 | 0.96 | 3.67 | 1.46 | 52.24 | 47.49 | 56.94 | 1.29 | 2.12 | 1.12 | 3.98 | 1.33 | 41.65 | 37.06 | 46.39 | 1.25 |
| 17 | Danguilan, et al. (2010) | 14.41 | 11.49 | 17.92 | 1.26 | 10.04 | 7.61 | 13.14 | 1.30 | 0.00 | 0.00 | 0.83 | 1.47 | 31.00 | 26.94 | 35.38 | 1.29 | 44.54 | 40.05 | 49.12 | 1.33 | 0.00 | 0.00 | 0.83 | 1.26 |
| 18 | Disthabanchong, et al (2018) | 19.31 | 14.96 | 24.55 | 1.25 | 10.04 | 6.94 | 14.30 | 1.28 | 0.00 | 0.00 | 1.46 | 1.38 | 60.62 | 54.55 | 66.37 | 1.27 | 4.63 | 2.67 | 7.92 | 1.30 | 5.41 | 3.25 | 8.87 | 1.25 |
| 19 | Duong, et al. (2015) | 30.09 | 22.40 | 39.09 | 1.23 | 30.97 | 23.19 | 40.01 | 1.23 | 2.65 | 0.91 | 7.52 | 1.16 | 23.89 | 16.97 | 32.53 | 1.23 | 9.73 | 5.52 | 16.59 | 1.23 | 2.65 | 0.91 | 7.52 | 1.23 |
| 20 | Goh, et al. (2002) | 58.82 | 49.84 | 67.26 | 1.23 | 4.20 | 1.81 | 9.46 | 1.23 | 2.52 | 0.86 | 7.15 | 1.17 | 33.61 | 25.76 | 42.50 | 1.23 | 0.84 | 0.15 | 4.61 | 1.23 | 0.00 | 0.00 | 3.13 | 1.23 |
| 21 | Griva, et al. (2017) | 25.96 | 20.77 | 31.92 | 1.25 | 9.36 | 6.26 | 13.77 | 1.28 | 8.09 | 5.24 | 12.28 | 1.36 | 28.94 | 23.51 | 35.04 | 1.27 | 0.00 | 0.00 | 1.61 | 1.29 | 0.00 | 0.00 | 1.61 | 1.25 |
| 22 | Griva, et al. (2018) | 10.53 | 6.58 | 16.41 | 1.24 | 1.97 | 0.67 | 5.64 | 1.25 | 3.95 | 1.82 | 8.34 | 1.25 | 75.66 | 68.26 | 81.79 | 1.25 | 7.89 | 4.57 | 13.29 | 1.25 | 0.00 | 0.00 | 2.46 | 1.24 |
| 23 | Hieu, et al. (2019) | 5.61 | 3.16 | 9.77 | 1.25 | 9.69 | 6.29 | 14.64 | 1.27 | 4.08 | 2.08 | 7.85 | 1.31 | 76.02 | 69.58 | 81.46 | 1.26 | 4.59 | 2.43 | 8.50 | 1.28 | 0.00 | 0.00 | 1.92 | 1.25 |
| 24 | Htun, et al. (2020) | 12.96 | 6.42 | 24.42 | 1.19 | 53.70 | 40.61 | 66.31 | 1.13 | 0.00 | 0.00 | 6.64 | 0.89 | 7.41 | 2.92 | 17.55 | 1.15 | 16.67 | 9.02 | 28.74 | 1.09 | 9.26 | 4.02 | 19.91 | 1.20 |
| 25 | Hyodo, et al. (2022) | 8.11 | 5.83 | 11.17 | 1.26 | 46.68 | 41.89 | 51.54 | 1.30 | 0.00 | 0.00 | 0.94 | 1.45 | 12.04 | 9.23 | 15.56 | 1.29 | 33.17 | 28.77 | 37.88 | 1.32 | 0.00 | 0.00 | 0.94 | 1.25 |
| 26 | Ingsathit, et al. (2010) | 7.83 | 6.80 | 9.00 | 1.27 | 10.97 | 9.75 | 12.31 | 1.32 | 0.00 | 0.00 | 0.17 | 1.58 | 24.50 | 22.78 | 26.30 | 1.31 | 5.61 | 4.74 | 6.63 | 1.36 | 47.91 | 45.87 | 49.96 | 1.26 |
| 27 | Ingsathit, et al.(2013) | 6.03 | 2.95 | 11.93 | 1.23 | 12.93 | 8.00 | 20.24 | 1.23 | 0.00 | 0.00 | 3.21 | 1.17 | 40.52 | 32.03 | 49.62 | 1.23 | 0.00 | 0.00 | 3.21 | 1.22 | 0.00 | 0.00 | 3.21 | 1.23 |
| 28 | Jalalonmuhali, et al. (2020) | 12.12 | 7.07 | 20.00 | 1.23 | 14.14 | 8.61 | 22.35 | 1.21 | 0.00 | 0.00 | 3.74 | 1.11 | 34.34 | 25.73 | 44.12 | 1.22 | 10.10 | 5.58 | 17.60 | 1.20 | 21.21 | 14.31 | 30.26 | 1.23 |
| 29 | Jonny, et al. (2020) | 32.17 | 26.47 | 38.46 | 1.25 | 28.26 | 22.84 | 34.40 | 1.27 | 0.00 | 0.00 | 1.64 | 1.35 | 33.48 | 27.70 | 39.80 | 1.27 | 4.78 | 2.69 | 8.36 | 1.29 | 0.00 | 0.00 | 1.64 | 1.25 |
| 30 | Koniman, et al. (2020) | 55.71 | 51.40 | 59.93 | 1.26 | 10.06 | 7.75 | 12.95 | 1.30 | 0.00 | 0.00 | 0.74 | 1.49 | 26.89 | 23.24 | 30.87 | 1.29 | 7.35 | 5.40 | 9.93 | 1.33 | 0.00 | 0.00 | 0.74 | 1.26 |
| 31 | Lee,et al. (2017) | 54.32 | 50.60 | 57.99 | 1.26 | 9.37 | 7.42 | 11.76 | 1.31 | 3.31 | 2.22 | 4.92 | 1.52 | 18.88 | 16.14 | 21.96 | 1.30 | 0.00 | 0.00 | 0.55 | 1.34 | 16.14 | 13.59 | 19.06 | 1.26 |
| 32 | Lew, et al. (2017) | 60.34 | 56.96 | 63.63 | 1.26 | 16.55 | 14.16 | 19.24 | 1.31 | 2.19 | 1.39 | 3.43 | 1.53 | 15.45 | 13.14 | 18.08 | 1.30 | 4.87 | 3.59 | 6.56 | 1.35 | 0.61 | 0.26 | 1.42 | 1.26 |
| 33 | Liu, et al. (2007) | 57.36 | 53.38 | 61.24 | 1.26 | 8.43 | 6.47 | 10.91 | 1.31 | 0.00 | 0.00 | 0.63 | 1.50 | 9.26 | 7.20 | 11.83 | 1.30 | 9.92 | 7.78 | 12.56 | 1.34 | 15.04 | 12.41 | 18.11 | 1.26 |
| 34 | Loy, et al. (2013) | 58.24 | 56.93 | 59.53 | 1.27 | 9.14 | 8.40 | 9.93 | 1.32 | 0.00 | 0.00 | 0.07 | 1.60 | 21.85 | 20.78 | 22.96 | 1.31 | 8.07 | 7.37 | 8.81 | 1.37 | 2.71 | 2.31 | 3.17 | 1.26 |
| 35 | Lumlertgul, et al. (2017) | 32.94 | 29.53 | 36.55 | 1.26 | 21.43 | 18.52 | 24.65 | 1.31 | 0.00 | 0.00 | 0.56 | 1.52 | 14.29 | 11.87 | 17.10 | 1.30 | 0.00 | 0.00 | 0.56 | 1.34 | 18.80 | 16.06 | 21.90 | 1.26 |
| 36 | Luvira, et al. (2011) | 42.64 | 37.44 | 48.01 | 1.26 | 14.71 | 11.31 | 18.92 | 1.29 | 0.00 | 0.00 | 1.14 | 1.42 | 12.61 | 9.47 | 16.61 | 1.28 | 8.11 | 5.63 | 11.54 | 1.32 | 0.00 | 0.00 | 1.14 | 1.25 |
| 37 | Malaysian Dialysis and Transplant Registry (2021) | 19.23 | 12.02 | 29.33 | 1.22 | 15.38 | 9.03 | 24.99 | 1.19 | 1.28 | 0.23 | 6.91 | 1.03 | 20.51 | 13.04 | 30.75 | 1.19 | 23.08 | 15.13 | 33.56 | 1.16 | 20.51 | 13.04 | 30.75 | 1.22 |
| 38 | Marbun, et al. (2017 | 31.16 | 24.03 | 39.31 | 1.24 | 26.81 | 20.12 | 34.76 | 1.24 | 0.00 | 0.00 | 2.71 | 1.22 | 16.67 | 11.37 | 23.77 | 1.24 | 13.04 | 8.41 | 19.68 | 1.24 | 12.32 | 7.84 | 18.84 | 1.24 |
| 39 | Maulidya, et al. (2022) | 8.75 | 4.30 | 16.98 | 1.22 | 55.00 | 44.12 | 65.42 | 1.19 | 0.00 | 0.00 | 4.58 | 1.04 | 16.25 | 9.75 | 25.84 | 1.20 | 13.75 | 7.85 | 22.97 | 1.16 | 0.00 | 0.00 | 4.58 | 1.22 |
| 40 | Mok, et al. (2012) | 1.78 | 0.90 | 3.47 | 1.26 | 2.89 | 1.70 | 4.88 | 1.30 | 4.22 | 2.72 | 6.50 | 1.47 | 73.33 | 69.06 | 77.21 | 1.29 | 17.78 | 14.52 | 21.58 | 1.33 | 0.00 | 0.00 | 0.85 | 1.26 |
| 41 | Ng, et al. (2012) | 84.62 | 70.27 | 92.75 | 1.17 | 0.00 | 0.00 | 8.97 | 1.08 | 0.00 | 0.00 | 8.97 | 0.76 | 0.00 | 0.00 | 8.97 | 1.10 | 15.38 | 7.25 | 29.73 | 1.01 | 0.00 | 0.00 | 8.97 | 1.18 |
| 42 | Noppakun, et al. (2015) | 8.51 | 7.68 | 9.42 | 1.27 | 12.81 | 11.81 | 13.89 | 1.32 | 0.00 | 0.00 | 0.10 | 1.60 | 23.91 | 22.61 | 25.26 | 1.31 | 7.50 | 6.72 | 8.36 | 1.37 | 47.29 | 45.75 | 48.85 | 1.26 |
| 43 | Ong, et al. (2017) | 44.26 | 32.51 | 56.70 | 1.20 | 27.87 | 18.19 | 40.17 | 1.15 | 0.00 | 0.00 | 5.92 | 0.93 | 21.31 | 12.90 | 33.12 | 1.17 | 6.56 | 2.58 | 15.68 | 1.11 | 0.00 | 0.00 | 5.92 | 1.21 |
| 44 | Paloyo, et al. (2022) | 10.34 | 3.58 | 26.39 | 1.14 | 6.90 | 1.91 | 21.96 | 1.01 | 3.45 | 0.61 | 17.18 | 0.64 | 79.31 | 61.61 | 90.15 | 1.04 | 0.00 | 0.00 | 11.70 | 0.92 | 0.00 | 0.00 | 11.70 | 1.15 |
| 45 | Panaput, et al. (2022) | 29.48 | 25.26 | 34.09 | 1.26 | 20.15 | 16.54 | 24.32 | 1.30 | 1.23 | 0.53 | 2.84 | 1.45 | 7.62 | 5.42 | 10.61 | 1.29 | 12.78 | 9.88 | 16.37 | 1.26 | 28.50 | 24.33 | 33.07 | 1.25 |
| 46 | Phang, et al. (2020) | 57.22 | 50.05 | 64.09 | 1.25 | 7.49 | 4.51 | 12.17 | 1.26 | 0.00 | 0.00 | 2.01 | 1.30 | 25.13 | 19.46 | 31.81 | 1.26 | 5.88 | 3.32 | 10.22 | 1.27 | 4.28 | 2.18 | 8.21 | 1.24 |
| 47 | Phongphithakchai, et al. (2022) | 13.57 | 8.86 | 20.23 | 1.24 | 0.00 | 0.00 | 2.67 | 1.24 | 5.00 | 2.44 | 9.96 | 1.22 | 30.71 | 23.67 | 38.79 | 1.24 | 3.57 | 1.53 | 8.09 | 1.24 | 46.43 | 38.37 | 54.67 | 1.24 |
| 48 | Premasathian, et al. (2010) | 5.82 | 4.36 | 7.72 | 1.26 | 9.92 | 7.99 | 12.26 | 1.31 | 0.00 | 0.00 | 0.51 | 1.52 | 11.77 | 9.67 | 14.27 | 1.30 | 0.00 | 0.00 | 0.51 | 1.35 | 65.87 | 62.42 | 69.16 | 1.26 |
| 49 | Rashid, et al. (2021) | 84.13 | 76.75 | 89.48 | 1.24 | 3.97 | 1.71 | 8.95 | 1.24 | 0.00 | 0.00 | 2.96 | 1.19 | 7.94 | 4.37 | 13.99 | 1.24 | 1.59 | 0.44 | 5.60 | 1.23 | 1.59 | 0.44 | 5.60 | 1.24 |
| 50 | Rudiansyah, et al. (2021) | 13.40 | 8.00 | 21.59 | 1.23 | 61.86 | 51.91 | 70.90 | 1.21 | 2.06 | 0.57 | 7.21 | 1.11 | 15.46 | 9.60 | 23.96 | 1.22 | 7.22 | 3.54 | 14.15 | 1.20 | 0.00 | 0.00 | 3.81 | 1.23 |
| 51 | Saengpanit, et al. (2017) | 52.00 | 38.51 | 65.20 | 1.19 | 8.00 | 3.15 | 18.84 | 1.12 | 0.00 | 0.00 | 7.13 | 0.85 | 6.00 | 2.06 | 16.22 | 1.14 | 16.00 | 8.34 | 28.51 | 1.07 | 18.00 | 9.77 | 30.80 | 1.20 |
| 52 | Sangadji, et al. (2020) | 39.39 | 24.68 | 56.32 | 1.15 | 54.55 | 37.99 | 70.16 | 1.04 | 0.00 | 0.00 | 10.43 | 0.69 | 0.00 | 0.00 | 10.43 | 1.07 | 6.06 | 1.68 | 19.61 | 0.96 | 0.00 | 0.00 | 10.43 | 1.17 |
| 53 | Santika, et al. (2021) | 32.25 | 27.27 | 37.67 | 1.26 | 59.61 | 54.03 | 64.95 | 1.29 | 1.95 | 0.90 | 4.20 | 1.41 | 1.30 | 0.51 | 3.30 | 1.28 | 4.89 | 2.98 | 7.90 | 1.31 | 0.00 | 0.00 | 1.24 | 1.25 |
| 54 | Segasothy, et al. (1986) | 4.44 | 2.27 | 8.52 | 1.25 | 5.00 | 2.65 | 9.23 | 1.26 | 2.22 | 0.87 | 5.57 | 1.29 | 33.33 | 26.86 | 40.50 | 1.26 | 11.67 | 7.76 | 17.18 | 1.27 | 43.33 | 36.31 | 50.64 | 1.24 |
| 55 | Seng, et al. (2018) | 49.37 | 45.24 | 53.51 | 1.26 | 12.39 | 9.91 | 15.38 | 1.30 | 1.62 | 0.85 | 3.04 | 1.49 | 11.31 | 8.94 | 14.21 | 1.29 | 5.21 | 3.65 | 7.38 | 1.34 | 20.11 | 16.99 | 23.64 | 1.26 |
| 56 | Shahar, et al. (2021) | 72.57 | 65.53 | 78.64 | 1.25 | 15.43 | 10.83 | 21.52 | 1.26 | 0.00 | 0.00 | 2.15 | 1.29 | 0.00 | 0.00 | 2.15 | 1.26 | 12.00 | 7.98 | 17.65 | 1.27 | 0.00 | 0.00 | 2.15 | 1.24 |
| 57 | Siagian, et al. (2018) | 20.28 | 15.42 | 26.20 | 1.25 | 71.23 | 64.80 | 76.90 | 1.27 | 0.94 | 0.26 | 3.37 | 1.33 | 6.13 | 3.62 | 10.21 | 1.27 | 1.42 | 0.48 | 4.08 | 1.29 | 0.00 | 0.00 | 1.78 | 1.25 |
| 58 | Suandewi, et al. (2020) | 18.18 | 11.15 | 28.24 | 1.22 | 29.87 | 20.80 | 40.85 | 1.18 | 3.90 | 1.33 | 10.84 | 1.02 | 6.49 | 2.81 | 14.32 | 1.19 | 41.56 | 31.21 | 52.71 | 1.16 | 0.00 | 0.00 | 4.75 | 1.22 |
| 59 | Supasyndh, et al. (2009) | 9.72 | 5.88 | 15.66 | 1.24 | 7.64 | 4.32 | 13.16 | 1.25 | 0.00 | 0.00 | 2.60 | 1.23 | 40.97 | 33.28 | 49.14 | 1.24 | 0.00 | 0.00 | 2.60 | 1.25 | 32.64 | 25.52 | 40.66 | 1.24 |
| 60 | Supit, et al. (2019) | 26.11 | 20.55 | 32.55 | 1.25 | 37.44 | 31.07 | 44.27 | 1.27 | 0.00 | 0.00 | 1.86 | 1.32 | 14.78 | 10.55 | 20.31 | 1.26 | 21.67 | 16.56 | 27.84 | 1.28 | 0.00 | 0.00 | 1.86 | 1.25 |
| 61 | Surendra, et al. (2019) | 46.10 | 38.08 | 54.32 | 1.24 | 22.70 | 16.56 | 30.28 | 1.24 | 4.96 | 2.43 | 9.89 | 1.23 | 17.02 | 11.71 | 24.08 | 1.24 | 0.00 | 0.00 | 2.65 | 1.25 | 9.22 | 5.47 | 15.14 | 1.24 |
| 62 | Suwan. (2011) | 31.21 | 24.78 | 38.46 | 1.25 | 87.86 | 82.16 | 91.92 | 1.26 | 0.00 | 0.00 | 2.17 | 1.28 | 22.54 | 16.95 | 29.33 | 1.26 | 21.39 | 15.93 | 28.08 | 1.27 | 0.00 | 0.00 | 2.17 | 1.24 |
| 63 | Tan, et al. (2014) | 6.12 | 2.10 | 16.52 | 1.19 | 4.08 | 1.13 | 13.71 | 1.12 | 0.00 | 0.00 | 7.27 | 0.85 | 46.94 | 33.70 | 60.62 | 1.13 | 6.12 | 2.10 | 16.52 | 1.06 | 36.73 | 24.67 | 50.73 | 1.20 |
| 64 | Tan, et al. (2019) | 42.72 | 33.60 | 52.36 | 1.23 | 15.53 | 9.79 | 23.75 | 1.22 | 0.97 | 0.17 | 5.30 | 1.13 | 24.27 | 17.02 | 33.38 | 1.22 | 8.74 | 4.67 | 15.78 | 1.21 | 7.77 | 3.99 | 14.58 | 1.23 |
| 65 | Tan, et al. (2020) | 47.28 | 41.64 | 52.98 | 1.26 | 16.67 | 12.84 | 21.35 | 1.29 | 4.42 | 2.60 | 7.42 | 1.40 | 23.47 | 18.99 | 28.64 | 1.28 | 8.16 | 5.55 | 11.86 | 1.31 | 0.00 | 0.00 | 1.29 | 1.25 |
| 66 | Tan. (2014) | 57.03 | 52.71 | 61.25 | 1.26 | 21.09 | 17.78 | 24.84 | 1.30 | 0.00 | 0.00 | 0.74 | 1.49 | 9.96 | 7.66 | 12.86 | 1.29 | 3.91 | 2.54 | 5.96 | 1.33 | 8.01 | 5.96 | 10.68 | 1.26 |
| 67 | Tang, et al. (2019) | 24.56 | 15.23 | 37.10 | 1.20 | 36.84 | 25.52 | 49.82 | 1.14 | 3.51 | 0.97 | 11.92 | 0.91 | 19.30 | 11.13 | 31.34 | 1.16 | 3.51 | 0.97 | 11.92 | 1.10 | 12.28 | 6.08 | 23.25 | 1.21 |
| 68 | Teo, et al. (2019) | 2.45 | 1.41 | 4.24 | 1.26 | 6.95 | 5.02 | 9.56 | 1.30 | 3.89 | 2.50 | 5.99 | 1.48 | 80.78 | 77.05 | 84.02 | 1.29 | 4.09 | 2.66 | 6.23 | 1.33 | 1.84 | 0.97 | 3.46 | 1.26 |
| 69 | Tng, et al. (2020) | 54.32 | 50.60 | 57.99 | 1.26 | 9.37 | 7.42 | 11.76 | 1.31 | 3.31 | 2.22 | 4.92 | 1.52 | 18.88 | 16.14 | 21.96 | 1.30 | 14.12 | 11.73 | 16.91 | 1.34 | 0.00 | 0.00 | 0.55 | 1.26 |
| 70 | Vareesangthip. (2017) | 26.59 | 23.21 | 30.27 | 1.26 | 23.41 | 20.19 | 26.97 | 1.31 | 2.34 | 1.40 | 3.89 | 1.50 | 14.38 | 11.80 | 17.42 | 1.29 | 14.55 | 11.95 | 17.60 | 1.34 | 18.73 | 15.81 | 22.05 | 1.26 |
| 71 | Viboon, et al. (2018) | 14.29 | 7.95 | 24.34 | 1.21 | 7.14 | 3.09 | 15.66 | 1.17 | 7.14 | 3.09 | 15.66 | 0.99 | 20.00 | 12.30 | 30.82 | 1.18 | 2.86 | 0.79 | 9.83 | 1.14 | 48.57 | 37.25 | 60.05 | 1.22 |
| 72 | Wei, et al. (2021) | 67.30 | 61.42 | 72.69 | 1.25 | 25.86 | 20.94 | 31.47 | 1.28 | 0.38 | 0.07 | 2.12 | 1.38 | 0.38 | 0.07 | 2.12 | 1.27 | 3.80 | 2.08 | 6.86 | 1.30 | 2.28 | 1.05 | 4.89 | 1.25 |
| 73 | Wong, et al. (2016) | 48.28 | 35.93 | 60.84 | 1.20 | 24.14 | 14.96 | 36.53 | 1.15 | 0.00 | 0.00 | 6.21 | 0.91 | 0.00 | 0.00 | 6.21 | 1.16 | 24.14 | 14.96 | 36.53 | 1.10 | 0.00 | 0.00 | 6.21 | 1.21 |
| 74 | Wong, et al. (2022) | 50.78 | 46.16 | 55.39 | 1.26 | 12.75 | 9.97 | 16.16 | 1.30 | 2.91 | 1.71 | 4.91 | 1.47 | 14.77 | 11.78 | 18.35 | 1.29 | 10.51 | 8.00 | 13.70 | 1.33 | 8.28 | 6.06 | 11.20 | 1.26 |
| 75 | Wongpraparut, et al. (2021) | 10.77 | 5.32 | 20.60 | 1.21 | 4.62 | 1.58 | 12.71 | 1.16 | 3.08 | 0.85 | 10.54 | 0.96 | 44.62 | 33.17 | 56.66 | 1.17 | 36.92 | 26.23 | 49.08 | 1.13 | 0.00 | 0.00 | 5.58 | 1.21 |
| 76 | Yap, et al. (2018) | 54.06 | 50.30 | 57.78 | 1.26 | 8.71 | 6.82 | 11.08 | 1.31 | 2.51 | 1.57 | 3.98 | 1.51 | 18.61 | 15.86 | 21.72 | 1.30 | 16.10 | 13.52 | 19.06 | 1.34 | 0.00 | 0.00 | 0.56 | 1.26 |
| 77 | Yusop, et al. (2013) | 22.22 | 14.87 | 31.85 | 1.22 | 7.78 | 3.82 | 15.19 | 1.20 | 0.00 | 0.00 | 4.09 | 1.08 | 14.44 | 8.64 | 23.16 | 1.21 | 11.11 | 6.15 | 19.26 | 1.18 | 33.33 | 24.45 | 43.58 | 1.23 |
| 78 | Zakaria, et al. (2021) | 52.89 | 47.49 | 58.22 | 1.26 | 13.98 | 10.65 | 18.15 | 1.29 | 2.13 | 1.03 | 4.33 | 1.42 | 4.86 | 3.02 | 7.75 | 1.28 | 15.20 | 11.72 | 19.48 | 1.31 | 10.94 | 8.01 | 14.78 | 1.25 |
| 79 | Zamri, et al. (2021) | 8.00 | 4.64 | 13.46 | 1.24 | 34.00 | 26.90 | 41.90 | 1.25 | 0.00 | 0.00 | 2.50 | 1.24 | 26.00 | 19.64 | 33.56 | 1.25 | 32.00 | 25.06 | 39.83 | 1.25 | 0.00 | 0.00 | 2.50 | 1.24 |
| 80 | Zukiman, et al. (2017) | 65.24 | 58.17 | 71.70 | 1.25 | 14.97 | 10.57 | 20.79 | 1.26 | 0.00 | 0.00 | 2.01 | 1.30 | 15.51 | 11.02 | 21.38 | 1.26 | 4.28 | 2.18 | 8.21 | 1.27 | 0.00 | 0.00 | 2.01 | 1.24 |
| 81 | Zukmin, et al. (2017) | 64.57 | 59.43 | 69.40 | 1.26 | 21.43 | 17.45 | 26.03 | 1.29 | 0.00 | 0.00 | 1.09 | 1.43 | 0.00 | 0.00 | 1.09 | 1.28 | 9.71 | 7.04 | 13.27 | 1.32 | 0.00 | 0.00 | 1.09 | 1.25 |
| **Overall random pooled ES** | | **29.18** | **23.88** | **34.78** | **100.00** | **16.78** | **14.05** | **19.70** | **100.00** | **0.74** | **0.40** | **1.16** | **100.00** | **20.01** | **16.84** | **23.38** | **100.00** | **8.64** | **6.97** | **10.47** | **100.00** | **7.54** | **4.32** | **11.50** | **100.00** |

**Supplementary Figure S2. Proportion of diabetic kidney disease across studies**

*Proportion 29.18%, 95%CI 23.88 – 34.78

*Significant heterogeneity between study: I^2^ = 99.12%; p<0.001

**Supplementary Figure S3. Proportion of hypertensive nephrosclerosis across studies**

*Proportion 16.78%, 95%CI 14.05 – 19.70

*Significant heterogeneity between study: I^2^ = 97.68%; p<0.001

**Supplementary Figure S4. Proportion of polycystic kidney disease across studies**

*Proportion 0.74%, 95%CI 0.40 – 1.15

*Significant heterogeneity between study: I^2^ = 89.68%; p<0.001

**Supplementary Figure S5. Proportion of glomerulonephritis across studies**

*Proportion 20.01%, 95%CI 16.84 – 23.38

*Significant heterogeneity between study: I^2^ = 98.04%; p<0.001


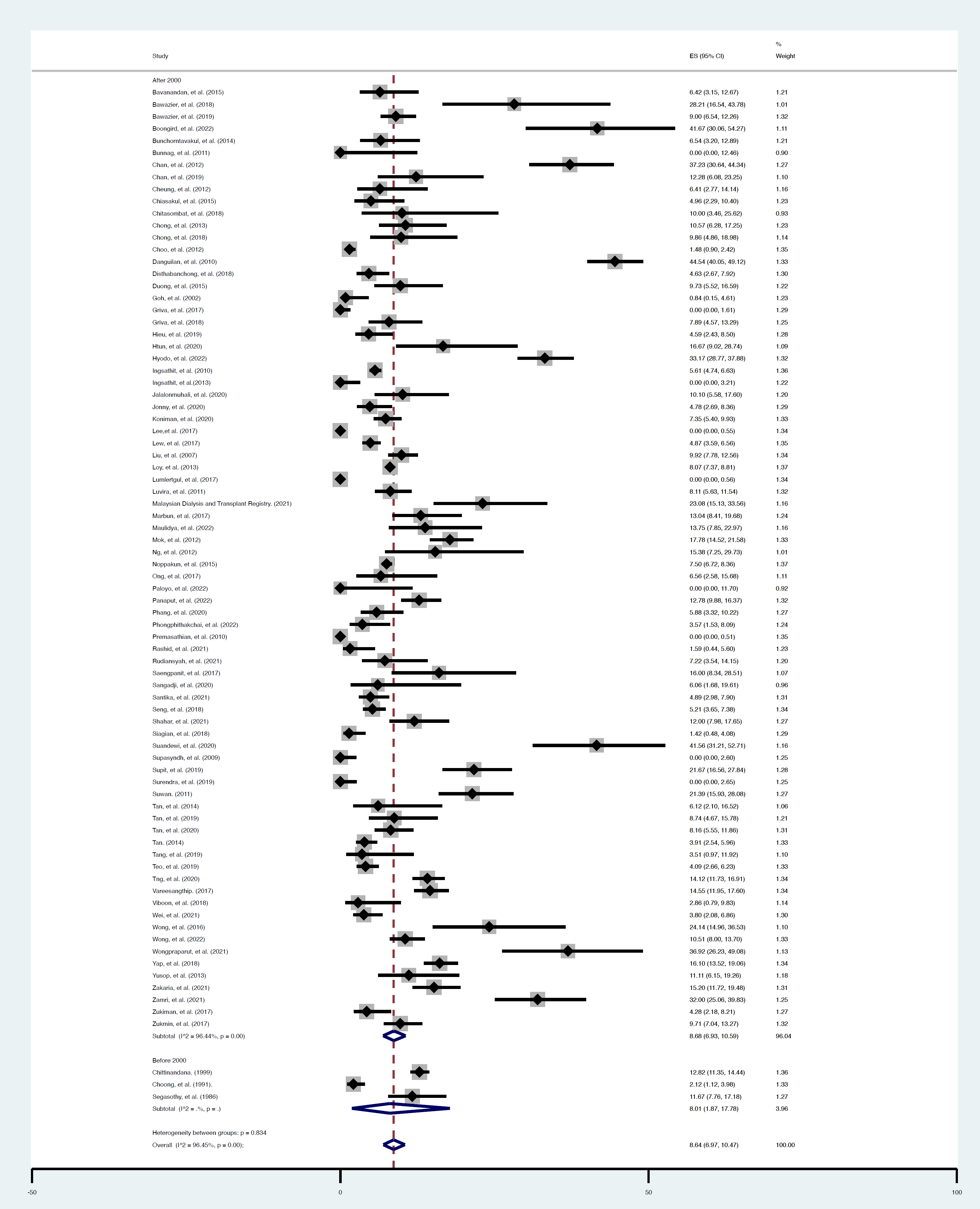


**Supplementary Figure S6. Proportion of other etiology across studies**

*Proportion 8.64%, 95%CI 6.97 – 10.47

*Significant heterogeneity between study: I^2^ = 96.45%; p<0.001

**Supplementary Figure S7. Proportion of unknown etiology across studies**

*Proportion 7.54%, 95%CI 4.32 – 11.50

*Significant heterogeneity between study: I^2^ = 99.29%; p<0.001

**Supplementary Figure S8. Sensitivity analysis on the proportion of diabetic kidney disease**

*Proportion 29.87%, 95%CI 21.97 – 38.41

*Significant heterogeneity between study: I^2^ = 98.96%; p<0.001

**Supplementary Figure S9. Sensitivity analysis on the proportion of hypertensive nephrosclerosis**

*Proportion 13.36%, 95%CI 9.34 – 17.94

*Significant heterogeneity between study: I^2^ = 97.84%; p<0.001

**Supplementary Figure S10. Sensitivity analysis on the proportion of polycystic kidney disease**

*Proportion 0.96%, 95%CI 0.38 – 1.74

*Significant heterogeneity between study: I^2^ = 90.26%; p<0.001

**Supplementary Figure S11. Sensitivity analysis on the proportion of glomerulonephritis**

*Proportion 19.38%, 95%CI 14.73 – 24.49

*Significant heterogeneity between study: I^2^ = 97.75%; p<0.001

**Supplementary Figure S12. Sensitivity analysis on the proportion of other etiology**

*Proportion 6.61%, 95%CI 4.27 – 9.38

*Significant heterogeneity between study: I^2^ = 96.60%; p<0.001

**Supplementary Figure S13. Sensitivity analysis on the proportion of unknown etiology**

*Proportion 13.57%, 95%CI 6.82 – 22.11

*Significant heterogeneity between study: I^2^ = 99.33%; p<0.001

**Supplementary Figure S14. Proportion of diabetic kidney disease across country**

*Proportion 29.18%, 95%CI 23.88 – 34.78

*Significant heterogeneity between study: I^2^ = 99.12%; p<0.001

**Supplementary Figure S15. Proportion of hypertensive nephrosclerosis across country**

*Proportion 16.78%, 95%CI 14.05 – 19.70

*Significant heterogeneity between study: I^2^ = 97.68%; p<0.001

**Supplementary Figure S16. Proportion of polycystic kidney disease across country**

*Proportion 0.74%, 95%CI 0.40 – 1.16

*Significant heterogeneity between study: I^2^ = 89.60%; p<0.001

**Supplementary Figure S17. Proportion of glomerulonephritis across country**

*Proportion 20.01%, 95%CI 16.84 – 23.38

*Significant heterogeneity between study: I^2^ = 98.04%; p<0.001

**Supplementary Figure S18. Proportion of other etiology across country**

*Proportion 8.64%, 95%CI 6.97 – 10.47

*Significant heterogeneity between study: I^2^ = 96.45%; p<0.001

**Supplementary Figure S19. Proportion of unknown etiology across country**

*Proportion 7.54%, 95%CI 4.32 – 11.50

*Significant heterogeneity between study: I^2^ = 99.29%; p<0.001

**Supplementary Figure S20. Proportion of diabetic kidney disease across study period**

**Supplementary Figure S21. Proportion of hypertensive nephrosclerosis across study period**

**Supplementary Figure S22. Proportion of polycystic kidney disease across study period**

**Supplementary Figure S23. Proportion of glomerulonephritis across study period**

**Supplementary Figure S24. Proportion of other etiology across study period**

**Supplementary Figure S25. Proportion of unknown etiology across study period**
